# Fast and inexpensive visualization of genome collection at scale

**DOI:** 10.1186/s12859-026-06498-w

**Published:** 2026-07-22

**Authors:** Sujatha Kotte, Naina Tiwari, Kavya Vaddadi, Vangala Govindakrishnan Saipradeep, Thomas Joseph, Aditya Rao, Rajgopal Srinivasan, Naveen Sivadasan

**Affiliations:** TCS Research, Tata Consultancy Services (TCS) Ltd, Hyderabad, India

**Keywords:** Genome atlas, Data visualization, Low dimensional embedding, SARS-CoV-2, BOVIZ

## Abstract

**Background:**

Investigating genetic progression and lineage using sequence data is a fundamental focus within the realm of viral genome research. Given the substantial increase in publicly accessible genome sequences, tools designed for extensive data analytics, particularly insightful visualizations play an important role in elucidating the spatio-temporal evolution as well as the diversity of viral lineages. Existing genome data analysis methods frequently rely on multiple sequence alignment (MSA) and phylogeny computations, making integration of new sequences computationally expensive. Such methods are often metadata agnostic and typically utilize a global distance metric between candidate sequences. We present BOVIZ, a fast and inexpensive method for rich visualization of large-scale genome collections. BOVIZ leverages variant-level features from candidate sequences and employs a novel Bag-Of-Variants (BOV) method that transforms high-dimensional variant feature vectors into a 2D-space, effectively capturing sequence (dis)similarities. BOVIZ circumvents the need for expensive MSA and phylogeny analyses, enabling efficient integration of novel sequences and faster visualization of large-scale sequences on a standard desktop. Furthermore, BOVIZ supports a variety of filters, including metadata filters and selection of genomic regions of interest for effectively capturing localized similarities and dissimilarities on-the-fly. We perform extensive benchmarking of BOVIZ with state-of-the-art visualization methods using the SARS-CoV-2 viral genome dataset.

**Results:**

BOVIZ consistently outperformed these methods in capturing inter-sequence similarity and diversity, offering superior visualization of spatio-temporal and clade-level evolution. It is able to effectively distinguish SARS-CoV-2 lineages into distinct clusters, visualize the evolution of specific sublineages like Omicron and their variant patterns, as well as visualize potential sub-cluster within the SARS-CoV-2 Delta variant.

**Conclusion:**

BOVIZ is an effective visualization tool for genome surveillance, complementing resource-intensive deep analytics by providing key insights about the underlying genome landscape in a fast and scalable manner.

## Background

Despite advancements in detection, epidemiology, treatment, and vaccines, viral diseases remain a persistent global challenge. Viruses such as Human Immunodeficiency Virus (HIV), Hepatitis C, Ebola, Measles, Severe Acute Respiratory Syndrome Coronavirus (SARS-CoV), Zika, Middle East Respiratory Syndrome Coronavirus (MERS-CoV), Dengue, and more recently severe acute respiratory syndrome coronavirus 2 (SARS-CoV-2) continue to pose significant threats to public health worldwide. The rapid evolutionary changes of viruses is a significant reason that hampers disease management efforts. Understanding these genomic changes is crucial for effective planning and response to viral outbreaks. Recent advances in next-generation sequencing (NGS) has resulted in a substantial increase in comprehensive human and viral genome datasets.

Platforms such as the Global initiative on sharing all influenza data (GISAID) [1] and 2019 Novel Coronavirus Resource (2019nCoVR), now known as RCoV19 [2], provide extensive viral genome data, including variant information and metadata. Therefore, there is a pressing need for efficient methods to analyze these datasets at scale, including intuitive visual analytics tools that complement other deeper analyses. Spatial-temporal visualization based on viral genomes offers insights into various aspects of viral diseases, such as origin of viral outbreaks, temporal patterns, transmission factors, clade characteristics, viral adaptation, pathogenicity as well as factors influencing the spread of the various strains of the virus [3, 4].

There are several methods for performing deeper analysis of the viral genomes, often based on MSA, phylogenetic analysis and haplotype networks. Phylogenetic analysis finds evolutionary relationships among organisms [5]. It discovers relationships between an ancestral sequence and its descendants. Haplotype network construction [6] is another well-used method to analyze as well as visualize relationships among genomic sequences with reference to a population or species. Haplotype networks are inferred using all identified haplotypes wherein, the reference sequence haplotype is taken as the starting node and based on the inheritance of mutations, its relationship with other haplotypes is computed. Hozumi et al. [7] demonstrated the application of UMAP-assisted K-means clustering to large-scale SARS-CoV-2 mutation datasets for viral genome clustering. Li et al. [8], in their study, compared four different methods and concluded that a deep learning clustering algorithm exhibited superior performance in clustering SARS-CoV-2 strains. Additional tools valuable in the analysis of SARS-CoV-2 include Infectious Pathogen Detector 2.0 (IPD 2.0) [9, 10], which facilitates genomic analysis to examine the variability and dynamics of the virus; IdCOV [11], identifies clades of SARS-CoV-2 virus isolates from raw sequencing data by utilizing a predefined list of clade-defining markers; and the COVID-19 CoV Genetics tool (COVID-19 CG) [12], which tracks SARS-CoV-2 SNVs, lineages, and clades of the virus genome with various filters. There is also a framework for genetic subtyping of SARS-CoV-2. It utilizes mutational signatures known as Informative Subtype Markers (ISMs) [13]. Additionally, Haplotype Explorer [14] enables the visualization and exploration of the virus genome using haplotype networks. All these tools and many others contribute in different ways to the understanding of the varied aspects of the SARS-CoV-2 like the origin, evolution and transmission, among others [15].

Visualizations offered by existing tools come with multiple drawbacks. Due to challenges in scalability, visualizations are often restricted to small subsets of data. spread.gl [16] is a feature-rich browser application which explores environmental factors that may have impacted pathogen dispersal patterns and helps interpret the complex outcomes of phylogeographic reconstructions. Nextstrain [17] and MicroReact [18] have also attempted to address some of the drawbacks in a limited fashion. Nextstrain supports visualization of phylogenetic and phylogeographic analyses using an animation similar to the SpreaD3 visualization application [19]. Often such tools use the expensive MSA and phylogeny studies to make meaningful visual representation and for exploration of genomic data. Furthermore, MSA and phylogeny analysis of a whole collection of viral genome is metadata agnostic, where inter-sequence distances are computed at a global scale. Such approaches lack the required specificity for users aiming to visually explore and analyze strain sub-collections or sub-sequences of interest based on filters from metadata or regions of interest. In such situations, it is desirable to have visualizations where users may seek to comprehend subsets of sequences according to their local (dis)similarities.

Principal Component Analysis (PCA), Uniform Manifold Approximation and Projection (UMAP), RadViz and t-distributed stochastic neighbor embedding (t-SNE) are widely popular methods for data visualization. PCA [20] identifies lower dimensional linear embedding while preserving majority of the variance in the data. t-distributed stochastic neighbor embedding (t-SNE) [21] is extensively utilized in the field of bioinformatics. While t-SNE can effectively capture local structure by reducing data dimensions, studies have reported inaccuracies in representing the global structure of the data [22]. UMAP [23] offers notably increased speed and better preservation of data’s global structure. RadViz [24] on the other hand, is a technique for visualizing multivariate d-dimensional data by positioning d anchor points evenly along the circumference of a 2D circle, with each data point connected to all dd anchor points via springs whose tensions correspond to the coordinate values. While techniques like UMAP and t-SNE are able to compartmentalize the data based on hyperparameters, they often lack the ability to adequately capture inter cluster and intra cluster (dis)similarities simultaneously. Capturing these (dis)similarities is crucial for visualizing and deriving meaningful insights from the spatiotemporal patterns present in the evolution of the strains.

We have developed a computationally efficient and scalable method, BOVIZ, to visualize and explore the spatiotemporal patterns present in large scale genome data. BOVIZ efficiently extracts variant level features of the strains and computes a novel and efficient Bag Of Variants (BOV) embedding to a lower dimensional space allowing the visualizations to effectively capture the similarity as well as divergence in the data. It can also support a variety of filters based on both the genomic regions of interest and the metadata such as geography, time interval and clade details. Taking the SARS-CoV-2 virus as a use case, we found BOVIZ performed better in capturing inter-sequence similarity and diversity, resulting in a more effective visualization of both spatio-temporal and clade-level evolution. It distinctly highlights similarities and differences among SARS-CoV-2 lineages as distinct clusters and effectively illustrates the evolution of specific sublineages and their variant patterns. This capability was demonstrated in the analysis of the Omicron variant as well as in visualizing a potential sub-cluster within the SARS-CoV-2 Delta variant. We believe that BOVIZ is an effective exploration and visualization tool that can aid in genome surveillance as well as complement other resource-intensive deep analytics tools in providing key insights about the underlying spatiotemporal landscape of the genome collection in a fast and scalable fashion.

## Methods

In this section, we present the details of our proposed embedding and visualization techniques.

Using the genotype vectors, we perform a novel low-dimensional non-linear embedding, named BOV (Bag Of Variants) embedding, inspired by the bag-of-visual-words embedding in computer vision. This in-turn is akin to the bag-of-words embedding in natural language processing (NLP). The approach is to construct clusters of related low-level features, called the vocabulary, for a given object collection. Using the vocabulary, a representation (embedding) is computed for the objects in the object collection, based on the occurrences of elements from the vocabulary. The object collection in our case is the set of genotype vectors and the vocabulary corresponds to the variant patterns that are observed in the sequence collection. The details are described below.

### Genotype vectors and BOV embedding

The input to our visualization pipeline consists of a set of genotype vectors, accompanied by relevant metadata, which correspond to the genome sequence collection. The genotype vectors encode the variants present in the sequence collection with respect to a reference sequence. These variants for each sequence can be computed through a one-time pairwise alignment with the reference sequence using tools such as Multiple Alignment using Fast Fourier Transform (MAFFT) [25]. The variant feature vector, in this case the genotype vector, represents a sequence as a binary vector, where each component is set to 1 only if the corresponding variant is present in the sequence. In this work, we mainly focus on single nucleotide variants (SNVs), as in most cases, they form the majority of all the detected variants. Formally, let $S= \{1, \ldots , n\}$ denote the input collection of *n* sequences. Let ${s_i} \in R^{m}$ denote the variant feature vector (genotype vector) corresponding to strain *i*. Here, *m* is the variant feature dimension. Both *n* and *m* are generally very large. In particular, the variant dimension *m* can be tens of thousands even for moderate sized genomes.

The initial step involves the construction of the vocabulary. To accomplish this, we perform a clustering of the sequences in S into k distinct clusters, based on the similarity of their variant features. This clustering yields a partition of *S* into the k clusters $S_1,...., S_k$, which constitutes our desired vocabulary. Specifically, the clusters are obtained using k-means clustering of the variant feature vectors of the sequences in S, with the Jaccard distance used to measure the similarity between the variant feature vectors. The Jaccard distance, which is computed between (binary) variant vectors, quantifies the similarity of the underlying variant sets of two sequences. Each cluster, therefore, represents a subset of sequences whose variant feature vectors exhibit similar latent variant patterns. These clusters, or the vocabulary, serve as the foundation for the subsequent non-linear embedding. The following section outlines efficient strategies for performing the desired clustering.

Efficient and scalable clustering for vocabulary construction: The direct application of the aforementioned clustering approach to the original input vectors can be computationally intensive, particularly given that the number of sequences, * n *, and the feature dimension, * m *, are typically large (on the order of tens of thousands). To enable efficient clustering that scales effectively with large sequence collections and high-dimensional feature spaces, we first perform a low-dimensional embedding of the genotype vectors prior to the clustering procedure. This approach is motivated by previous works [26, 27], which demonstrated that high-dimensional vectors can be effectively clustered by first projecting them into lower-dimensional spaces through data-oblivious embedding techniques. Specifically, it has been shown that k-means clustering performed in a lower-dimensional space with o(k) dimensions can approximate the solution obtained in the original higher-dimensional space. In particular, it was shown in [26] that for Euclidean *k*-means on *n* points, data oblivious random projections onto $O(\log (1/\epsilon ) (\log (k) + \log \log (n))/\epsilon ^6)$ dimensions would suffice to preserve the clustering solution within a multiplicative $(1 \pm \epsilon )$ factor. Further improvements were made in [27], where it was shown that for a broad class of random linear projections, termed sub-Gaussian-tailed random maps, $O(\log (k/\epsilon )/\epsilon ^2)$ dimensions are sufficient. Inspired by these results, we employ sparse random projection matrices [28] to project each of the $L_2$-normalized sequence feature vectors $s_i / ||s_i||$ for $\ i = {1\ldots n}$, to obtain the corresponding *d*-dimensional projected vectors $\{s'_1, \ldots , s'_n\}$ where *d ≪ m* is a chosen parameter. The use of sparse random projection matrices allows for efficient computation, even for large datasets, and these matrices are known to closely approximate random maps with Gaussian N(0,1) entries [28].

We now proceed to apply Euclidean k-means clustering on the d-dimensional projections. It is important to note that, as initially stated, the objective is to perform clustering based on Jaccard distance. Specifically, Jaccard distance is used to compare the variant feature vectors of the sequences. However, since Euclidean k-means clustering has been shown to effectively preserve the clustering solution even when applied to lower-dimensional projections (in our case, the d-dimensional vectors), we opt to use Euclidean k-means instead of directly employing Jaccard distance for the clustering procedure.

A formal justification for this choice is provided in Section A of the Supplementary File, where it is demonstrated that, under certain assumptions, the Euclidean distance between L2 -normalized sequence vectors serves as a reasonable approximation for the Jaccard distance between the original variant feature vectors. It is also noteworthy that the low-dimensional vectors $\{s'_1, \ldots , s'_n\}$ are solely used for the purpose of efficient k-means clustering and are not utilized in subsequent steps of the analysis. The output of the clustering process defines a partition of the original set S into k subsets, denoted as $S_1,...S_k$, which constitutes the desired vocabulary.

Following the computation of the *k* clusters $S_1,...S_k$ (vocabulary), the next step involves averaging the variant feature vectors within each cluster to compute the corresponding centroid vectors $\{c_1, \ldots c_k\}$. That is, $c_i = \frac{1}{|S_i|} \sum _{j \in S_i} s_j$. The final *k*-dimensional BOV embedding $bov: \mathbb {R}^m \rightarrow \mathbb {R}^k$ is computed using the weighted Jaccard distance between the sequence vectors and the *k* centroids. Recall that sequence vectors are binary and centroids are real valued. Weighted Jaccard between two *k*-dimensional vectors *u* and *v* is given by $WJD(u, v) = 1 - \frac{\sum _1^k \min (u_i, v_i)}{ \sum _1^k \max (u_i, v_i)}$ [29]. Using these distances, the final BOV embedding for a variant feature vector $s_i$ is given by

$$ bov(s_i) = (b_1, \ldots , b_k), $$

where the j-th component of the BOV embedding, $b_j$, is given by the weighted Jaccard distance between $s_i$ and the centroid $c_j$ of the j-th cluster:

$$ b_j = WJD(s_i,c_j) $$

Thus, each component of the BOV embedding represents the weighted Jaccard distance between the sequence’s variant feature vector and the centroid of the corresponding cluster. To obtain a 2D visualization of the sequences, standard Principal Component Analysis (PCA) is applied to the k-dimensional BOV embedding vectors of the sequences.

It is important to note that when computing the BOV embedding for strains based on selected regions of interest from the genome, only the coordinates of the variant feature vectors that lie within these regions are retained. The BOV embedding is then performed on these trimmed variant feature vectors.

### Visualization of the spatio-temporal landscape of SARS-CoV-2 genome sequences

We applied the BOVIZ method to visualize SARS-CoV-2 viral genome sequences, which are publicly available at [2]. For visualization, we initially employed standard Principal Component Analysis (PCA) and Radial Visualization (RadViz) [24] to visualize the BOVIZ embeddings. We then compared the BOVIZ visualizations with several conventional 2D visualization approaches, including PCA, UMAP, t-SNE, and Kernel PCA. In these comparisons, the variant feature vectors were used in place of the BOV vectors. Kernel Principal Component Analysis (KPCA) can work directly with the Gram matrix that captures pairwise dissimilarity between the data points. A natural choice for constructing the Gram matrix is the gapped alignment score between all pairs of strains. However, computing this for all pairs can be computationally expensive. To mitigate this, we approximated the pairwise dissimilarities by using distances derived from the variant feature vectors of the strains. Specifically, we used two different forms of distance measures, namely, the cosine distance (KPCA-Cosine) and the Jaccard distance (KPCA-Jaccard) between the variant feature vectors of strain pairs, for constructing different choices of Gram matrices.

To visualize the data points, we used color coding of the data points incorporating metadata information in the visualization. This will help in capturing the clade level, temporal and spatial (geographic) evolution of the data. Color coding for the spatial and clade level metadata are straightforward. However, color coding each distinct time point at the date level may lead to visual clutter and overload. Similarly, visualizing a large number of strain-level data points can result in a congested and difficult-to-interpret canvas. To address these challenges, when the dataset contains a large number of data points, we perform random sampling to reduce the number of points displayed, thereby improving the clarity of the visualization (Fig. 1). For temporal metadata analysis, we apply binning based on collection dates (e.g., by week, month, or other user-defined time periods). A heatmap indicating the corresponding temporal bin for each data point is used to enhance visualization and provide a clearer representation of temporal trends.

**Fig. 1:**
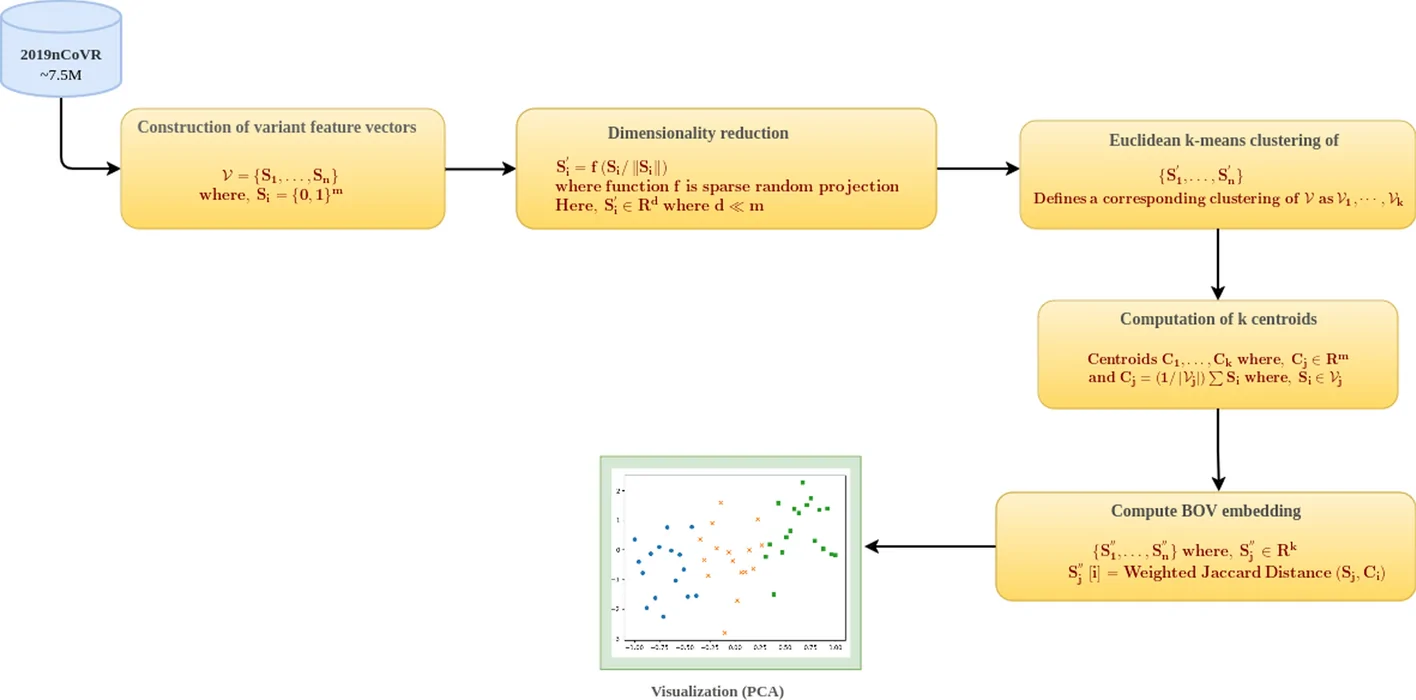
Schematic representation of BOVIZ visualization pipeline

### Implementation

The SARS-CoV-2 genome data were downloaded from the China National Center for Bioinformation/National Genomics Data Center which has created and maintains the 2019 Novel Coronavirus Resource (2019nCoVR) [2]. We downloaded the Variant Call Format (VCF) file for SARS-CoV-2 spanning the period from December 24, 2019, to May 4, 2023. This file contains 7,489,212 columns, with each column corresponding to a specific strain. The primary focus of this analysis is on single nucleotide variants (SNVs), which account for more than 90% of the variants described in the VCF file. The dataset includes 29,779 rows, each representing a variation site. The first nine columns of the VCF file provide metadata and information about the variant sites. Columns 1 through 3 specify the genomic position of each variation site, while column 4 denotes the reference allele of SARS-CoV-2, and column 5 lists the alternative allele. In cases where multiple alternative alleles are observed, the alleles are separated by commas in column 5. When the alternative allele is ambiguously sequenced, degenerate nucleotide symbols such as Y, W, and others are used. Columns 6 to 8 remain empty, and column 9 serves as an indication to users that the subsequent columns contain genotype information.

The vcf file is preprocessed in such a way that each row should contain one alternate allele. After the initial preprocessing steps, a total of 78,006 variant sites were obtained. The genomes in the VCF file correspond to the pathogen, and the associated metadata pertains to both the host and the pathogen. The metadata file contains 16 fields of which we utilize Host, Collection date, Location, Lineage metadata fields. These metadata attributes are explicitly utilized for color coding the genomes on the visualization canvas, though they are not incorporated into the input genotype vectors. Some metadata fields contain incomplete or missing information. Further preprocessing excludes entries in metadata with incomplete information and its corresponding genome. After this step, a total of 7,347,356 strains remained in the dataset. As the resulting vector is huge, there would be an increase in storage. Loading such high-dimensional data into memory can lead to long loading times and substantial computational overhead. However, since the variant feature vectors are sparse (where 0 indicates the absence of a variant and 1 indicates the variant position), we performed data compression by storing only the indices of the 1’s. This approach reduces the memory footprint and facilitates faster data loading.

We provide a Jupyter notebook that facilitates BOVIZ visualization of the resulting SARS-CoV-2 strain dataset. This notebook enables users to generate customized plots based on their strains of interest. Interactive widgets within the notebook allow users to select various parameters, including country, timeline, lineage, genomic region of interest, the maximum number of data points to display on the canvas, and the type of plot to generate.

Given the large number of samples from certain countries, the notebook includes a configurable limit, allowing users to select up to a maximum of 15k samples for analysis. This limitation is implemented to prevent overcrowding of the visualization canvas. Additionally, users can opt to color-code the resulting plots based on metadata fields such as Country, Pangolin Clade, and Week, thereby enhancing the interpretability of the visualizations.

### Quantitative benchmarking

We conducted a systematic benchmarking of BOVIZ against five alternative SOTA methods – Principal Component Analysis (PCA), Kernel PCA (KPCA), KPCA with random sparse projection (RSP), UMAP, and t-SNE – across five representative SARS-CoV-2 visualization scenarios. Run-1, Run-2, and Run-3 correspond to the spatio-temporal evolution of variants of concern (VOCs) in four countries (Brazil, India, South Africa, and the UK) for the years 2020, 2021, and 2022, respectively, as shown in Figs. 2, 3 and 4. Run-4 examines the distribution of Omicron (BA lineages) sequences from South Africa as shown in Fig. 5, while Run-5 focuses on the Delta (B.1.617.2) variant and its sub-lineages in India during 2021 as seen in Fig. 7. In each case, all methods were applied to the same input variant feature vectors. We selected the Saturn coefficient [30] as the primary evaluation metric, as it captures both local and global structure preservation in a single value. The Saturn score ranges from 0 (no preservation of original structure) to 1 (perfect preservation) and thus provides an intuitive yardstick for embedding quality on large genomic datasets.

The Saturn coefficient was chosen over classical metrics like trustworthiness (TW) and continuity (CN) because it provides a more holistic assessment of embedding quality [30]. The Saturn metric captures both local and global structures simultaneously by essentially comparing all pairwise distances in high-dimensional space vs. the 2D embedding. It is computed using the adjusted RV coefficient between the original and embedded distance matrices, yielding a single integrated score that reflects the balance of local and global structure retention. In practical terms, a high Saturn score indicates that both the fine-grained neighborhood relationships and the broader cluster configuration of the data are well preserved in the 2D plot.

To assess robustness to sampling variability, we performed 10-fold sampling for each benchmarking scenario (Runs 1–5). For each fold, an equal-sized subsample of strains was drawn using the same sampling protocol across all methods, and each method was evaluated on the corresponding subsample. This yields a distribution of embedding-quality measurements per (run, method), enabling us to quantify variability and avoid conclusions driven by a single sample realization.

**Effect of hyperparameters**: To ensure a fair and reproducible comparison between BOVIZ and state-of-the-art dimensionality reduction and visualization methods, we conducted all benchmarking experiments using carefully tuned hyperparameters for other methods used in benchmarking. For BOVIZ, no hyperparameter tuning needs to be performed. All methods were applied to the same variant–feature vectors derived from SARS-CoV-2 genome sequences, with no additional filtering or transformation. For each method and dataset (Runs 1–5), we performed hyperparameter tuning using grid search over commonly recommended ranges, selecting configurations that maximized structure preservation as measured by the Saturn coefficient.

For example, UMAP was evaluated with varying numbers of neighbours ($n_{\text {neighbors}} \in \{20, 30, 50, 100\}$), minimum distance ($\text {min\_dist} \in \{0.1, 0.25, 0.5, 0.8, 0.99\}$), and distance metrics (Cosine, Jaccard, Euclidean). Similarly, t-SNE was tuned over perplexity (20, 30, 50,100), learning rate (auto, 200, 300,500), and distance metrics (Cosine, Jaccard, Euclidean). KPCA and KPCA-RSP were evaluated with both linear kernel and Jaccard similarity kernel.

### Scalability profiling and resource efficiency

To evaluate the scalability of BOVIZ in comparison to the other benchmarked methods, we conducted a systematic profiling study across increasing dataset sizes, ranging from 10k to 1 million SARS-CoV-2 genome sequences, recording runtime and peak memory usage on a workstation (DELL Precision 7875 with AMD Ryzen Threadripper Pro processor, 512 GB RAM). All methods were executed in the same hardware environment to ensure comparability. For each method and dataset size, we recorded two key metrics: (i) peak memory usage (in GB) and (ii) total runtime (in seconds). The methods profiled include BOVIZ, Incremental PCA, Kernel PCA (KPCA), KPCA with Random Sparse Projection (KPCA-RSP), t-SNE, and UMAP.

### Interpreting bag-of-variants

The Bag-of-Variants (BoV) representation encodes each viral genome as a high-dimensional, sparse binary vector, where each dimension corresponds to the presence or absence of a specific genomic variant. We conducted additional experiments to investigate the underlying biological signals conserved within the BOVIZ clusters for a given dataset. To evaluate this, we applied a contrastive pattern mining (CPM) approach [31] to characterize the variant composition and specificity of BOV-derived clusters. To evaluate the biological and technical interpretability of the BOV representation, we applied CPM to characterize the variant composition of BOV-derived clusters using Run-4. We performed BOV interpretation on the 14-dimensional BOV vectors based on 14 clusters identified by BOVIZ. For each cluster,we computed internal and external conservation values as defined in Chen et al. [31] of each variant using CPM and derived a growth rate metric [31] to identify cluster-specific variant signatures. Variants with high internal conservation (>0.7) and low external conservation (<0.3) were retained and ranked by growth rate to prioritize those with the strongest cluster specificity.

## Results

In this section, we use BOVIZ to visualize SARS-CoV-2 strains, with the aim of deriving insights into their spatio-temporal evolution. Additionally, we provide a comparative analysis of BOVIZ visualizations alongside those generated using other standard visualization approaches. Supplementary plots and additional supporting material are provided in Section B of the Supplementary file to further illustrate the results.

### BOVIZ reveals clade and temporal patterns in SARS-CoV-2 variants

Several clinically significant variants of SARS-CoV-2 have emerged as a result of mutations, which may contribute to antigenic drift and enable the virus to evade host immune recognition. Variants of concern (VOCs) are specific genetic variations that possess evolutionary advantages and are favored in the evolutionary process. The major SARS-Cov-2 variants of concern (VOCs) include B.1.1.7 (Alpha), B.1.351 (Beta), B.1.617/B.1.617.2 (Delta), P.1 (Gamma) and BA.2 (Omicron). These VOCs have emerged during different time points. The appearance of B.1.1.7 in the United Kingdom (UK) was followed by B.1.351 in South Africa, B.1.617 in India, P.1 in Brazil, and BA in South Africa [32].

These VOCs independently originated from one another, quickly gaining dominance either regionally or globally, thereby outcompeting preceding variants. In this study, we aim to use BOVIZ to visually explore the evolution and transmission dynamics of different SARS-CoV-2 variants across various geographic regions over the course of the pandemic. To explore how different VOCs exhibited transmission advantage over each other during the relevant yearly time period (2020-2022), we have collectively visualized strains from UK, South Africa, India and Brazil for year 2020, 2021 and 2022 with default parameters of all lineages, genomic regions of interest and a limit of 10k samples on the canvas for each of the selected years. It may be recalled that the Alpha, Beta and Gamma VOCs were prevalent primarily in 2020, Delta in 2021 and Omicron in 2022.

**Fig. 2:**
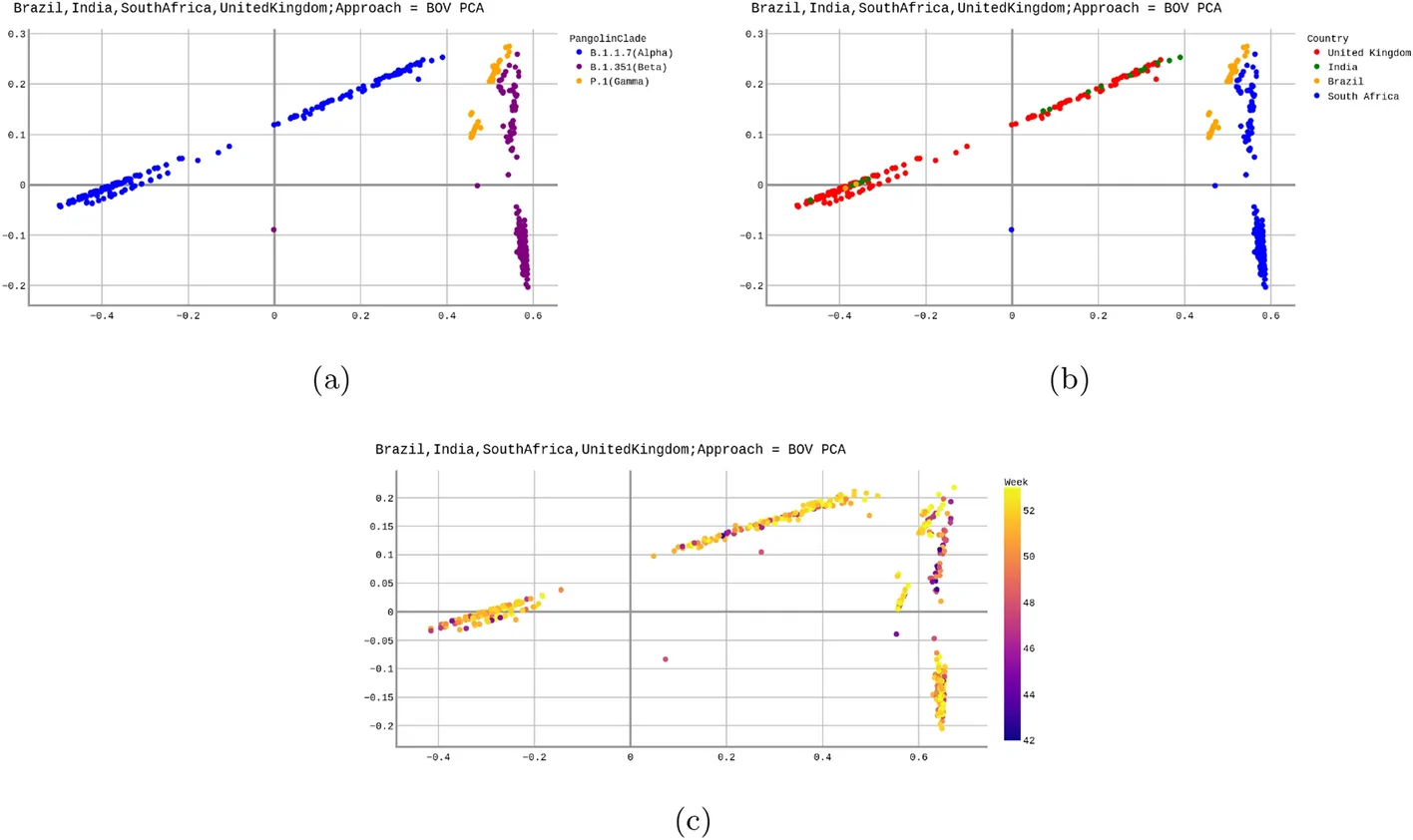
BOVIZ visualization of VOC strains from Brazil, India, South Africa and United Kingdom for the year 2020. Color coding is done with respect to **a** Pangolin Clade, **b** Geography and **c** temporal

**Fig. 3:**
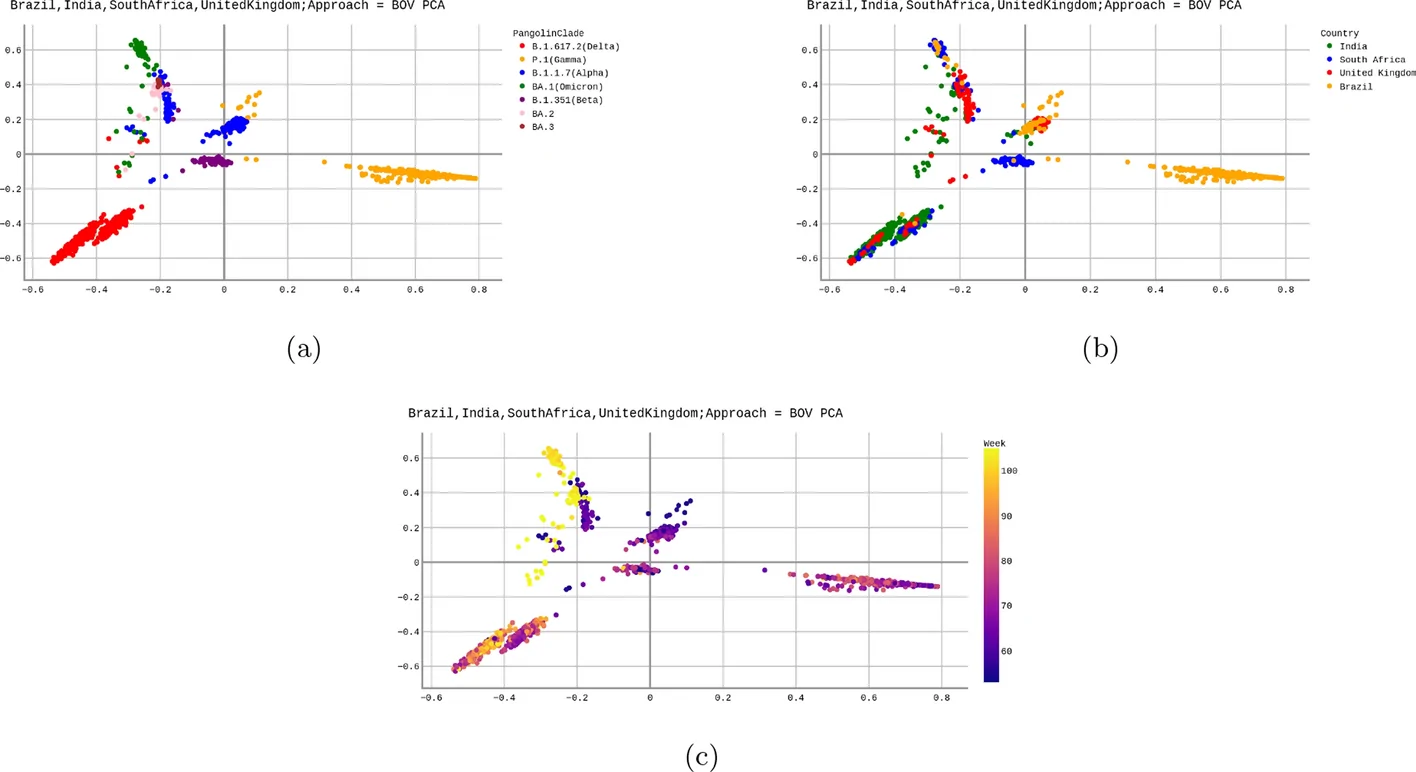
BOVIZ visualization of VOCs in Brazil, India, South Africa and United Kingdom for the year 2021. Color coding is done with respect to **a** Pangolin Clade, **b** Geography and **c** temporal

**Fig. 4:**
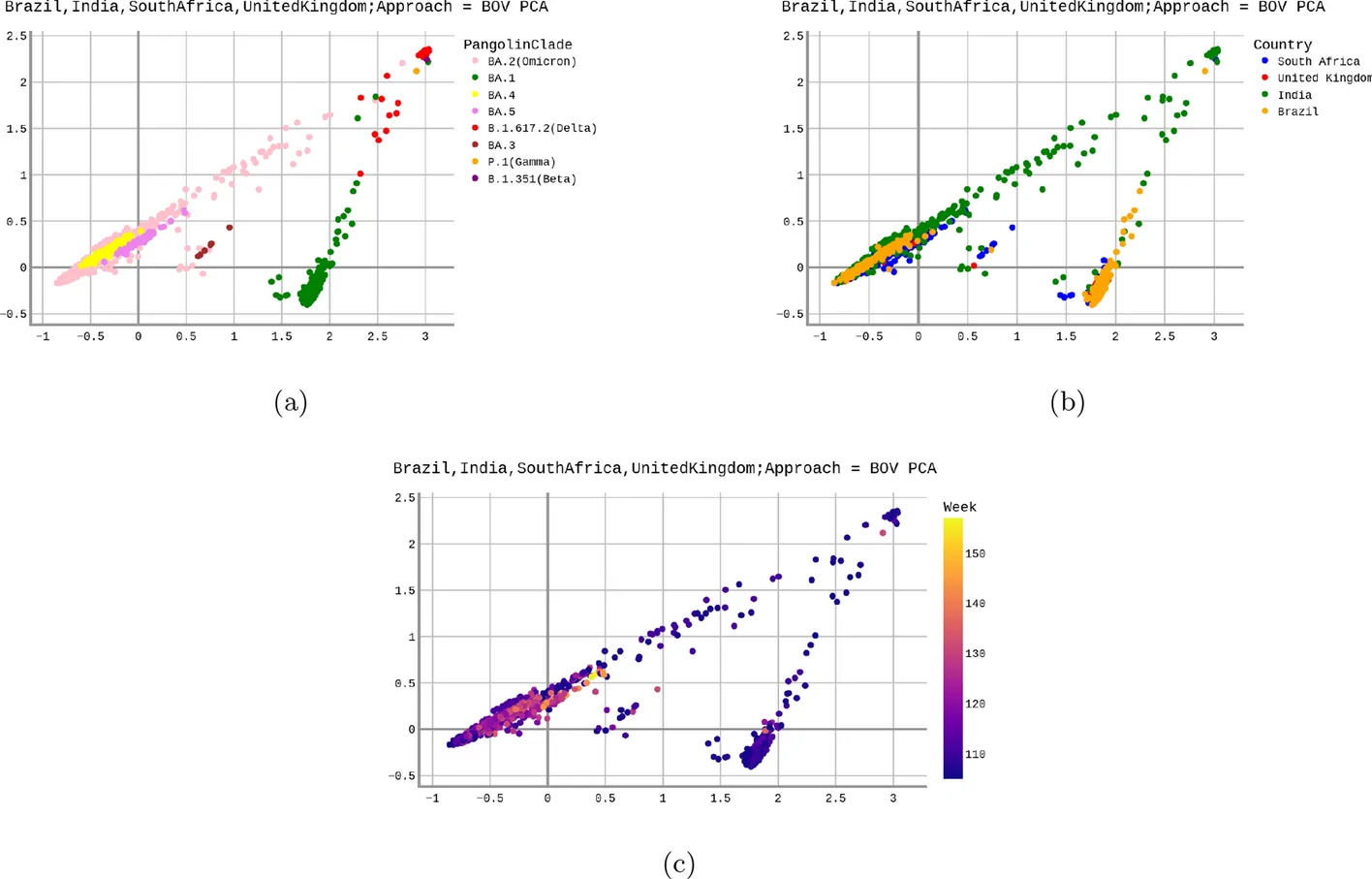
BOVIZ visualization of VOCs in Brazil, India, South Africa and United Kingdom for the year 2022. Color coding is done with respect to **a** Pangolin Clade, **b** Geography and **c** temporal

Figures 2, 3 and 4 present the BOVIZ visualizations of the VOC strains from Brazil, India, South Africa, and the United Kingdom for the years 2020, 2021, and 2022, respectively. Corresponding figure using t-SNE, UMAP and standard PCA are provided in Section B of the Supplementary file. Figure 2 illustrates the strains from 2020, which are primarily associated with the Alpha (B.1.1.7), Beta (B.1.351), and Gamma (P.1) variants. Figures 2a, 3a and 4a demonstrates that the clade level separation is well captured by BOVIZ visualization. Further, intra-clade separation suggests possible sub-variant clusters that have significantly different genomic composition. As a consequence, these subsets could potentially have significant separation from the remaining strains within the same clade as well as strains from other clades. BOVIZ captures this by spatial separation of these subsets in the visualization highlighting their markedly reduced genomic similarity with the remaining strains. Moreover, the spatial proximity of strains in Fig.2c reveals that several earlier strains from the Beta clade and later strains from the Alpha clade share greater similarity with Gamma strains. This pattern aligns with the global lockdowns that occurred in 2020, which restricted the spread of VOCs to the regions where they first emerged, as observed in Fig. 2b. However, as lockdown measures were lifted in subsequent years, the geographical distribution of VOCs expanded, as reflected in Figs. 3b and 4b. Notably, Fig. 3a shows the spatial proximity between the Alpha (B.1.1.7) and Omicron (BA.2) variants, which can be attributed to a significant percentage of sequence similarity between these two variants [33, 34]. For the year 2022, the dominating strains are from the Omicron family. BOVIZ clearly captures the Omicron sub-families as separate clusters as seen in Fig. 4a. Interestingly, the spatial proximity among the different sub-lineages reveals the higher relative similarity of the BA.2, BA.4 and BA.5 sub-lineages and their marked separation from the BA.1 sub-lineage. As reported in [35–37], BA.1 and BA.2 share multiple common mutations but also differ by few specific mutations respectively. Specifically, BA.1 and BA.2 have 13 and 8 unique mutations when compared against each other resulting in forming separate clusters as seen in Fig. 4a. Furthermore, BA.4 and BA.5 cluster closely with BA.2, likely due to similarities in the mutations present in the spike region of these variants. Here, we can also observe very few strains from BA.3 forming cluster in between BA.1 and BA.2 which can be supported with the fact that BA.3 spike protein does not have specific mutations but shares mutations between BA.1 and BA.2 [38]. Visualization using standard approaches on similar datasets are provided in the Supplementary materials.

Most visualizations focus on capturing compact, well separated clusters that accounts for intra-cluster similarity and inter-cluster separation. This often results in not capturing the inter-cluster relatedness and intra-cluster separation adequately. BOVIZ visualization captured the different clades as well-separated clusters and, at the same time, inter-cluster spatial proximity and intra-cluster spatial separation are also adequately captured.

### BOVIZ visualization of evolution of omicron strains and its comparison with standard approaches

Recent studies have demarcated the Omicron variant (B.1.1.529.1 or BA.1) into its sublineages such as BA.1.1 (B.1.1.529.1.1), BA.2 (B.1.1.529.2), and BA.3 (B.1.1.529.3), BA.4 (B.1.1.529.4) and BA.5 (B.1.1.529.5). These are the first five branches descending from the original Omicron ancestor [36]. BA.1, BA.2 and BA.3 are different from one another. Initially, BA.1 was the most prolific omicron sub-lineage detected worldwide. Later on, BA.2 overtook BA.1 as the dominant variant globally. BA.2 has 8 unique mutations, that are not found in BA.1 and lacks 13 mutations observed in BA.1. More recently, two new sub-lineages, BA.4 and BA.5, were discovered in South Africa. The spike from BA.4 and BA.5 are seen to be most closely related to BA.2 than others [35].

Figure 5 shows the BOVIZ visualization of the distribution of SARS-CoV-2 Omicron sequences from South Africa, from the onset of coronavirus disease 2019 (COVID19) till May 4, 2023. The lineages BA.1, BA.2 and BA.3 form three distinct clusters. BA.1 and BA.1.1 are seen grouped together in the visualization (Fig. 5a), as they share many common mutations [37]. Lineage BA.2 form a distinct cluster separate from BA.1 family, which is justified due to the fact that compared with BA.1 and BA.1.1, the spike protein mutations in BA.2 are very different [37]. Since the BA.4 and BA.5 lineages are known to be closely related to BA.2, we can observe a greater closeness between their respective clusters. Figure 5b shows the evolution of omicron variant with respect to time in weeks measured from the start of the pandemic in December 2019. Interestingly, as expected, the fact that the later strains of BA.2 have closer resemblance to the earlier strains of BA.4 and BA.5, is well captured in the visualization.

**Fig. 5:**
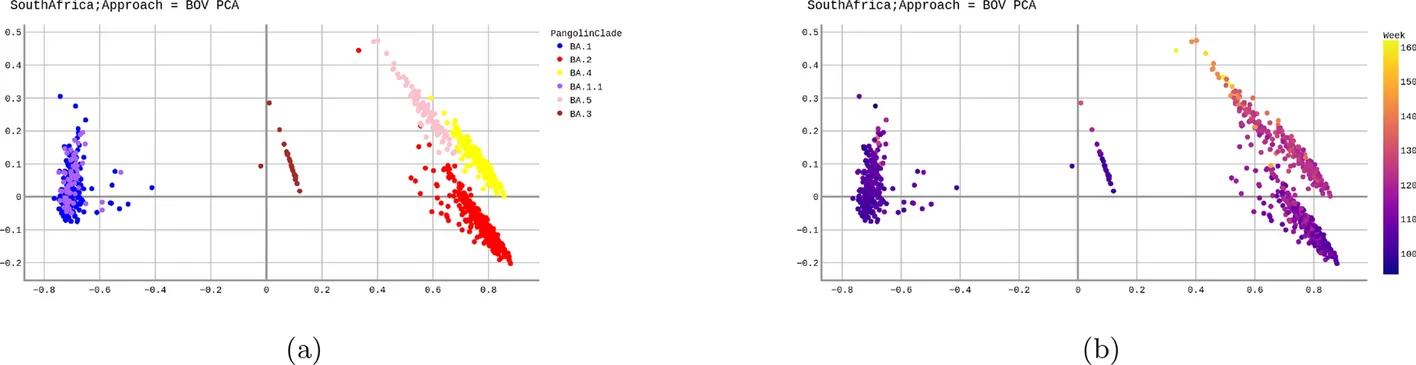
Omicron strains from South Africa. Visualizations using BOVIZ. The strains are colored based on their respective Pangolin clade in **a** and with respect to the timeline (reporting week) in **b**

For comparing the visualization quality of BOVIZ, we also considered the standard visualization methods such as the standard PCA, t-SNE and UMAP, applied to the strain level variant feature vectors. These visualizations are given in Fig. 6. These visualizations clearly demonstrate that BOVIZ is better in capturing the inherent data clusters, providing an effective and intuitive visualization of both temporal and clade-level evolution of the strains. As seen in Fig. 6, PCA (Fig. 6a) and UMAP visualizations 6b do not capture these clusters as effectively. When PCA was applied on variant feature embedding vector, we found that maximum variance captured by Standard PCA was relatively low as compared to the BOVIZ tool. Further, the intra-cluster and inter-cluster similarities captured by t-SNE are less useful in visualizing the overall clade level and temporal evolution of the strains.

**Fig. 6:**
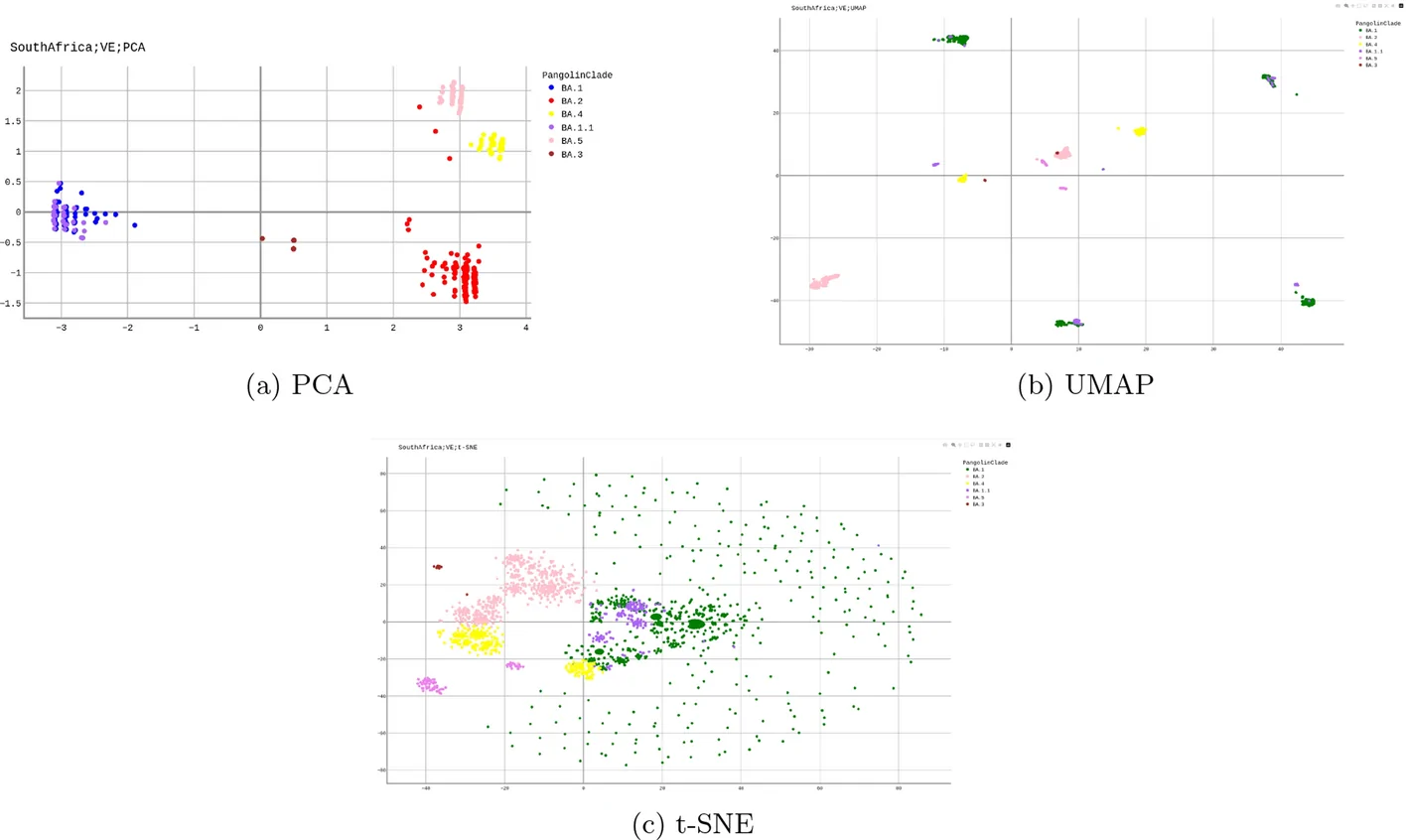
Visualization of Omicron strains from South Africa using the standard **a** PCA, **b** UMAP and **c** t-SNE visualizations applied to the strain level variant feature vectors (genotype vectors). Strains are colored based on their respective Pangolin clades

### BOVIZ uncovers a potential sub-cluster within the SARS-CoV-2 delta variant

The abrupt increase in COVID-19 cases in India during 2021, particularly the devastating second wave, aligns with the prevalence of the B.1.617.2 variant. The rise in Delta variant cases may be associated with its heightened transmissibility, capability to evade immune responses within the human body, and potential challenges in diagnostic detection [39]. Within VOCs, the delta variant played a significant role in increasing the severity and mortality of COVID-19, likely stemming from a decline in antibody affinity caused by viral antigenic mutations in the receptor-binding domain [40].

We visualized the delta strains collected in the year 2021 to get a snapshot of genomic variations among the delta sub-lineages. Figure 7a shows distinct clusters respective to each sub-lineages. Interestingly, it is found that delta (B.1.617.2) forms two arms which could be an indication of further mutations within B.1.617.2, leading to formation of two sub-clusters. The temporal plot, Figure 7b further reveals that the upper arm is formed by the samples collected from more recent dates than the lower arm. To further understand variations coming from specific regions of interest, we plotted the spike genomic region. As seen in Fig. 7c, this does not describe the distinct sub clusters seen in Fig. 7a. In addition to the numerous characteristic spike mutations observed in the Delta variant, a study by Banerjee et al. [41] identified a unique set of eight non-spike mutations that co-occur in samples collected from India. This report suggested a potential pattern of mutations in non-spike proteins, which could influence the evolution of a distinct sub-cluster within the Delta variant. Further investigation of these non-spike mutations revealed the presence of two distinct arms, as observed previously. Therefore, the presence of these non-spike mutations may provide an explanation for the formation of these two sub-clusters. BOVIZ is able to effectively highlight these distinctive mutations in a more accessible and intuitive manner.

**Fig. 7:**
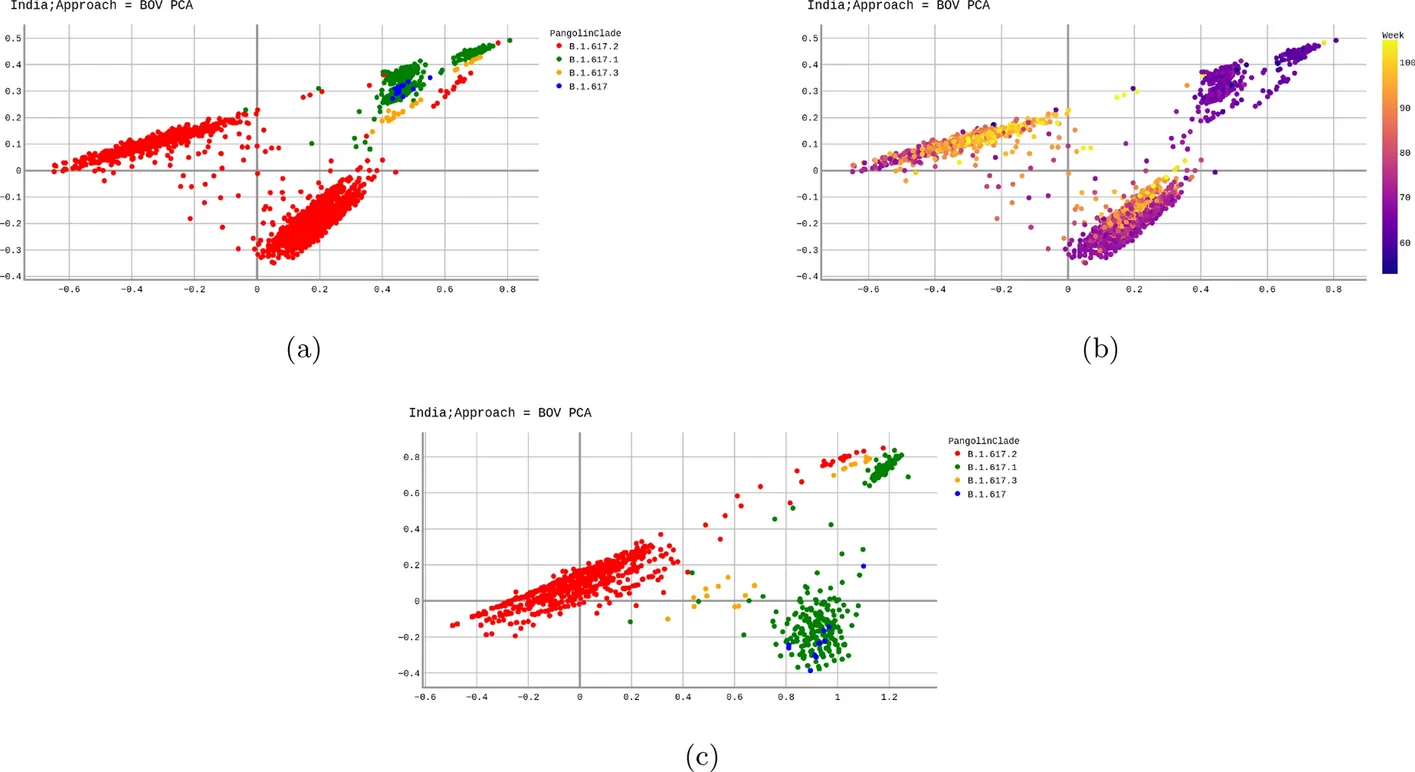
BOVIZ visualization of the Delta variant and its sub- lineages in India for year 2021, **a** whole genomic region; **b** week number-based distribution; **c** spike genomic region

### Quantitative benchmarking

Table 1 summarizes the Saturn scores (mean ± standard deviation) across 10 folds for each method and run. BOVIZ consistently achieves the highest average Saturn score across all scenarios, with relatively low dispersion, indicating both better and stable structure preservation of the original high-dimensional relationships in its 2D embeddings compared to alternative techniques in structural fidelity. This trend holds both relatively simple cases and more challenging scenarios with complex, heterogenous data. Notably, in Run-3, BOVIZ attained a Saturn of 0.158 - an order of magnitude greater than UMAP’s 0.003 and t-SNE’s 0.003 - reflecting BOVIZ’s ability to retain meaningful structure where standard methods essentially collapsed to an almost uninformative scatter. Similarly, for Run-4, BOVIZ yielded a Saturn of  0.17, roughly three times higher than PCA or KPCA ( 0.05) and over six-fold higher than UMAP (0.025). These quantitative gains align with our qualitative observations: BOVIZ plots showed well-defined, distinctive clusters with sensible inter-cluster proximities (e.g. BA.2 positioned between BA.1 and BA.4/BA.5 in the Omicron visualization), whereas competing methods often produced overlapping or uninterpretable embeddings (e.g. UMAP failed to separate certain clades, yielding near-zero Saturn scores). Even in Run-5 (Delta sub-lineages in India), where all methods struggled to some extent (Saturn <0.05, indicating the inherent difficulty of the task), BOVIZ still outperformed the others (0.044 vs.  0.02−0.03). The consistency of BOVIZ’s top-ranking Saturn scores across these diverse experiments is especially compelling: unlike PCA or t-SNE, which show highly variable performance depending on the dataset, BOVIZ provides reliably high structure preservation in every case (Table 1). This suggests that BOVIZ’s design confers a generalizable advantage in capturing genomic relationships, from broad spatio-temporal patterns (Runs 1-3) to detailed lineage/sublineage differentiation (Runs 4-5).

**Table 1:** Saturn scores (mean ± standard deviation) across 10 folds for each method and run

Run (dataset)	BOVIZ	PCA	KPCA$^{\textrm{a}}$	KPCA-RSP$^{\textrm{b}}$	UMAP	t-SNE
Run-1 (VOC 2020) Brazil/India/S. Africa/UK	**0.3607** ± 0.0253	0.3051 ± 0.0391	0.3051 ± 0.0391	0.1818 ± 0.0932	0.1442 ± 0.1428	0.0418 ± 0.0188
Run-2 (VOC 2021) Brazil/India/S. Africa/UK	**0.0860** ± 0.0192	0.0814 ± 0.0090	0.0815 ± 0.0090	0.0650 ± 0.0149	0.0781 ± 0.0286	0.0470 ± 0.0210
Run-3 (VOC 2022) Brazil/India/S. Africa/UK	**0.1582** ± 0.0159	0.0469 ± 0.0178	0.0469 ± 0.0178	0.0404 ± 0.0352	0.0034 ± 0.0020	0.0030 ± 0.0013
Run-4 (Omicron, South Africa)	**0.1663** ± 0.0285	0.0527 ± 0.0001	0.0527 ± 0.0001	0.0564 ± 0.0059	0.0251 ± 0.0121	0.0489 ± 0.0042
Run-5 (Delta, India 2021)	**0.0440** ± 0.0186	0.0311 ± 0.0043	0.0311 ± 0.0043	0.0286 ± 0.0191	0.0296 ± 0.0168	0.0209 ± 0.0081

^a^KPCA using variant-based cosine-distance kernel;^*b*^KPCA using Jaccard-distance kernel (approximated via sparse random projection)

The values in bold are the best Saturn scores across all methods and runs

**Effect of hyperparameters**: Supplementary Table 1 contains the best Saturn score achieved by the other benchmarked methods with optimal hyperparameter selection separately for each run. The BOVIZ execution remained the same without any hyperparameter selection. Here, BOVIZ achieved top scores in 4 out of 5 runs, where it achieved better scores than others in 3 runs and near similar score for 1 run. Note that for each of these runs, the best performing hyperparameter choices for other methods vary. While this is not a feasible approach for a user, the goal of this experiment was to evaluate the sensitivity of these methods to hyperparameter choices and to provide a transparent comparison of the methods.

This experiment demonstrates the use of BOVIZ as a practical large-scale or automated genomic visualization workflows with minimal user intervention. The consistent performance of BOVIZ across diverse datasets, without the need for per-run tuning, supports its robustness and ease of deployment in real-world settings.

**Scalability profiling and resource efficiency**: BOVIZ exhibits robust scalability, maintaining efficient runtime (Fig. 8a) and low peak memory usage (Fig. 8b) across increasing dataset sizes (Fig. 8). In contrast to methods like Incremental PCA, t-SNE, and UMAP—which require substantially more resources and become impractical at scale—BOVIZ remains efficient for large-scale genomic surveillance, processing millions of SARS-CoV-2 sequences without excessive hardware demands. Notably, BOVIZ remains the only method in our evaluation capable of processing all 7.34 million samples in the dataset with peak memory of 5.5 GB in 1163 s. Furthermore, running BOVIZ on 1 million samples using a HP laptop (with Intel Ultra 7 processor; 32GB) took 522 s with peak memory consumption of 5 GB.

**Fig. 8:**
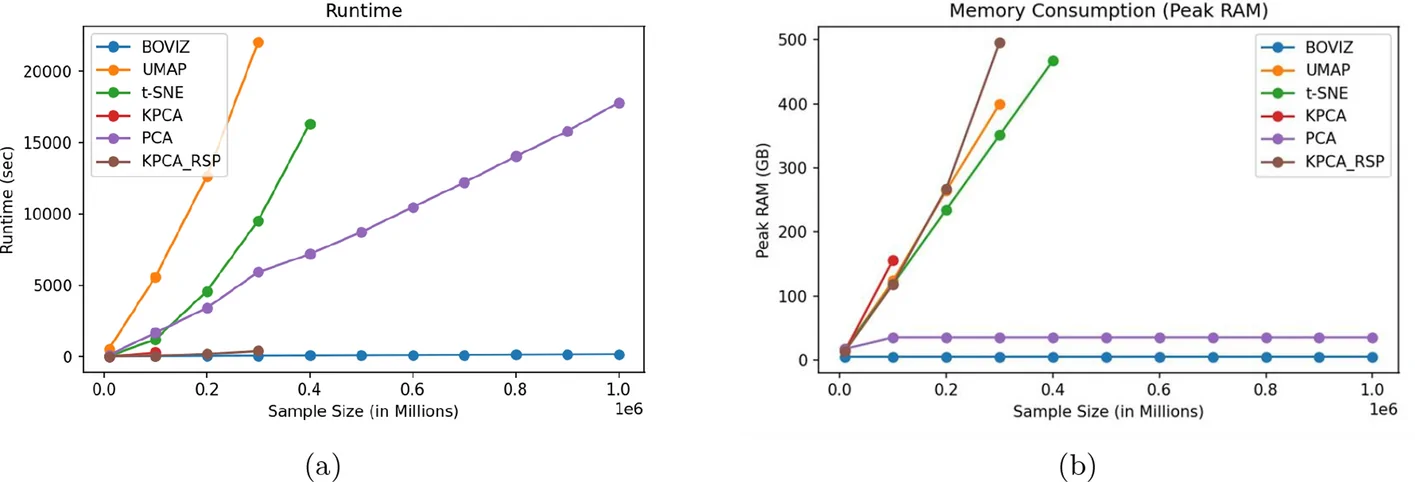
**a** Scalability Profiling, and **b** Resource Efficiency analysis

These results substantiate the scalability claims of BOVIZ. Its efficient runtime profile and low memory requirements enable efficient processing of large-scale genomic datasets, including those exceeding 7 million sequences. In contrast, standard methods such as t-SNE, UMAP, and KPCA exhibit prohibitive computational costs at scale.

### Interpreting bag-of-variants

The analysis revealed that BOV clusters are underpinned by distinct and biologically meaningful variant sets. Cluster 5 (n = 392) exhibited near-complete internal conservation (mean *≈ 0.99*), very low external conservation, and elevated growth rates—features consistent with adaptive or lineage-associated mutations. Variant analysis of this cluster revealed enrichment for Omicron-associated Spike protein substitutions, including L452R and F486V in the receptor-binding domain and P681H at the S1/S2 cleavage site. These mutations are known to confer antibody escape and enhance viral entry and have been observed across multiple BA.1, BA.2, BA.4, and BA.5 sub-lineages. Most variants in this cluster are annotated as having “moderate” predicted impact, consistent with adaptive changes that preserve protein function while enhancing viral fitness [42, 43].

Among other clusters of interest is cluster 7, which contains a consistent set of Spike mutations (e.g., G339D and N501Y) together with highly conserved non-Spike mutations such as ORF1ab:P4715L and N:R203K/G204R. While the Spike mutations reflect known Omicron antigenic changes, the conserved non-Spike mutations suggest stable genomic backbones [44, 45]. Cluster 0 contrasts with more conserved clusters by showing greater variability in its variant composition. Although it includes common Omicron background variants (S:D614G, N:R203K/G204R), these co-occur with a wide and heterogeneous set of Spike mutations (e.g., A67V, T95I, and N-terminal domain changes) [44, 46], as shown in Supplementary Table 2. Taken together, these findings demonstrate that the BOV clusters appear to capture functionally and evolutionarily coherent sub-populations, and that the structure of clusters in the embedded visualization reflects underlying evolutionary patterns rather than arbitrary binning.

## Discussion

In this study, we introduce a computationally efficient, scalable, and user-friendly visualization approach for analyzing large collections of viral genomes. Experiments show that our approach of expressing the inter-strain similarities of SARS-CoV-2 based on the BOV embedding is able to capture both the temporal dynamics and clade-level evolution of the virus. This approach provides valuable insights into the evolutionary patterns of SARS-CoV-2, presenting the data in a manner that is both informative and interpretable. The high dimensionality of variant feature spaces introduces challenges in accurately quantifying distances between strains, as pairwise distance measures that aggregate differences at the variant level often suffer from the curse of dimensionality. This leads to less informative pairwise distance metrics. However, to effectively reveal spatio-temporal patterns in viral strains, it is essential to identify latent variant patterns that are conserved across strains. In the context of latent variant patterns, pairwise distances typically exhibit heavy-tailed distributions, necessitating visualization methods that are specifically designed to accommodate such distributions. These methods should be capable of emphasizing similarities and dissimilarities in a manner that unveils spatio-temporal trends in the data. Computing latent variant patterns in real-time for interactive visualizations can pose computational challenges. BOVIZ adopts an efficient approach by utilizing underlying clusters (bags of variants) as proxies for latent variant patterns, facilitating on-the-fly computation. This method enables BOV embeddings of the strains to effectively capture the heavy-tailed nature of pairwise distances. Importantly, our visualization method does not rely on computationally expensive approaches, such as phylogenetic analysis or multiple sequence alignment (MSA) computations. Consequently, the BOVIZ approach is both scalable and capable of handling large-scale genomic datasets efficiently.

In this work, Pangolin annotations are used primarily as a familiar interpretability layer and a consistency check, not as ground truth evaluation. The contribution of BOVIZ lies in providing a scalable, alignment free visualization framework that enables exploration of continuous similarity structure, intra-lineage heterogeneity, and region-specific effects, which are not captured by discrete lineage labels alone. Further, our approach supports embedding and visualizations based on specific genomic regions of interest, which can be helpful in deeper understanding of the strain landscape. In order to observe and study the temporal level evolution of collected samples, we performed temporal binning on the samples, which gives much deeper insights into viral spread and evolution during the course of time. We can view a large number of samples by performing sampling so as to get a uniform distribution of samples through a given period of time. This gives better visualization of samples when plotting combination of regions together. In the future, we will focus on systematically mapping the conserved variant features identified by BOVIZ to genomic coordinates and gene annotations to improve biological interpretation of BOV embeddings. This will enable downstream gene-level and pathway-level analyses using established functional annotation resources. Linking cluster structure to such annotations may help distinguish biologically meaningful signals from background variation.

## Conclusion

The goal of BOVIZ was to provide a visual aid for analyzing large collections of viral genome sequences. Each sequence in the collection is a data point in the canvas. Our low dimensional embedding attempts to capture the sequence (dis)similarities. These visualizations can aid in further analysis to identify the landscape of the crucial genomic mutations that underline the observed divergences. Our visualizations do not directly identify these mutations but can serve as a valuable visual aid in this process. We believe that our visualization approach can provide useful visualizations for other metagenome and pangenome collections beyond the COVID-19 datasets. Augmenting the input feature vectors along with the metadata information could also be used in our overall approach for improved visualization performance.

## Additional file

Supplementary file 1.

## Data Availability

This work is licensed under the Creative Commons Attribution-NonCommercial-ShareAlike 4.0 International License CC BY-NC-SA 4.0. As a use case, this study used Variant Call Format (VCF) files for SARS-CoV-2 spanning the period from December 24, 2019, to May 4, 2023 - these are available from the China National Center for Bioinformation/National Genomics Data Center which has created and maintains the 2019 Novel Coronavirus Resource (2019nCoVR), now known as RCoV19 [2]. The BOVIZ code base is available at https://github.com/sujathakotte/BOVIZ.

## Abbreviations

2019nCoVR: 2019 Novel Coronavirus Resource
BOV: Bag-of-variants
COVID-19 CG: COVID-19 cov genetics
GISAID: Global initiative on sharing all influenza data
HIV: Human immunodeficiency virus
IPD: Infectious pathogen detector
ISM: Informative subtype marker
MAFFT: Multiple alignment using fast Fourier transform
MERS-CoV: Middle east respiratory syndrome coronavirus
MSA: Multiple sequence alignment
NGS: Next-generation sequencing
NLP: Natural language processing
PCA: Principal component analysis
RCoV19: Resource for coronavirus 2019
SARS-CoV: Severe acute respiratory syndrome coronavirus
SARS-CoV-2: Severe acute respiratory syndrome coronavirus 2
t-SNE: T-distributed stochastic neighbor embedding
UMAP: Uniform manifold approximation and projection
VCF: Variant call format
VOC: Variant of concern
