# Supplementary material for: Fast and inexpensive visualization of genome collection at scale
Source: BMC Bioinformatics. 2026 Jul 22;27:239. doi: 10.1186/s12859-026-06498-w (PMC13679107; doi:10.1186/s12859-026-06498-w)
Supplement: Supplementary file 1 [file 12859_2026_6498_MOESM1_ESM.pdf]

A Approximating Jaccard Distances

We show that under certain assumptions, Euclidean distances between l_2 normalized sequence feature vectors are reasonable approximations to Jaccard distances between the sequence feature vectors. We recall that the sequence vectors $\{s_1, \ldots, s_n\} \subset \mathbb{R}^m$ are binary vectors. That is, vector s_i encodes the subset of variants V_i that are present in the sequence i. Therefore, the normalized vector for s_i is given by $s_i/|V_i|$. Let $|V_i| = n_i$, $|V_j| = n_j$ and $|V_i \cap V_j| = c$. The Jaccard distance between two vectors s_i and s_j are given by

$$JD(s_i, s_j) = 1 - |V_i \cap V_j|/|V_i \cup V_j| \ = \ 1 - \frac{c}{n_i + n_j - c}$$

Let $s'_i = s_i/||s_i||_2$. The Euclidean distances between L_2-normalized vectors, denoted by $NED(s_i, s_j)$ is then given by

$$\begin{aligned} NED(s_i, s_j) = ||s'_i - s'_j||_2^2 \ &= \ \frac{n_i - c}{n_i} + \frac{n_j - c}{n_j} + c\left(\frac{1}{\sqrt{n_i}} + \frac{1}{\sqrt{n_j}}\right)^2 \ = \ 2 - \frac{2c}{\sqrt{n_i n_j}} \\ &= \ 2\left(1 - \frac{c}{\sqrt{n_i n_j}}\right) \end{aligned}$$

If we ignore the constant factor in the Euclidean distance, because it is the same for all pairwise distances, we obtain that $JD(s_i, s_j) = 1 - \frac{c}{n_i + n_j - c}$ and $NED(s_i, s_j) = \left(1 - \frac{c}{\sqrt{n_i n_j}}\right)$. If we focus on the SNP variants and consider the fact that the SNP variants can be assumed to be reasonably independent and random mutations from a large space of mutations, by the law of large numbers, the number of obseved SNPs in the sequences are similar. Hence, if we approximate $n_i \approx n_j \approx n'$, we obtain that $JD(s_i, s_j) \approx 1 - \frac{c}{n' + n' - c}$ and $NED(s_i, s_j) \approx 1 - \frac{c}{n'}$. That is, the additional term in the denominator of JD is $n' - c$. From these distance approximations, the corresponding similarities are given by $1 - JD \approx c/n'$ and $1 - NED \approx c/(2n' - c)$.

Now, suppose we consider a sequence s_i and its similarity (or distance) to two other sequences s_j and s_j. Let $c_j = |V_i \cap V_j|$ denote the number of common variants for s_i and s_j. Similarly, let $c_k = |V_i \cap V_k|$ denote the number of common variants for s_i and s_k. Here again, we make the approximating assumption that $|V_i| = |V_j| = |V_k|$. We can see that sequence s_i is more similar to s_j than s_k under JD if and only if s_i is more similar to s_j than s_k under NED. Clearly, the same statement holds for distances as well. Formally, $1 - JD(s_i, s_j) \leq 1 - JD(s_i, s_k)$ if and only if $1 - NED(s_i, s_j) \leq 1 - NED(s_i, s_k)$. Using the approximations for JD and NED, this corresponds to showing that $c_j/n' \leq c_k/n'$ if and only if $c_j/(2n' - c_j) \leq c_k/(2n' - c_k)$. This is straightforward to show. In conclusion, the ordering of sequence vectors based on distances is preserved under both JD and NED.

B Visualizations of the VOC strains from Brazil, India, South Africa, and the United Kingdom for the years 2020, 2021, and 2022 respectively using t-SNE, UMAP and standard PCA

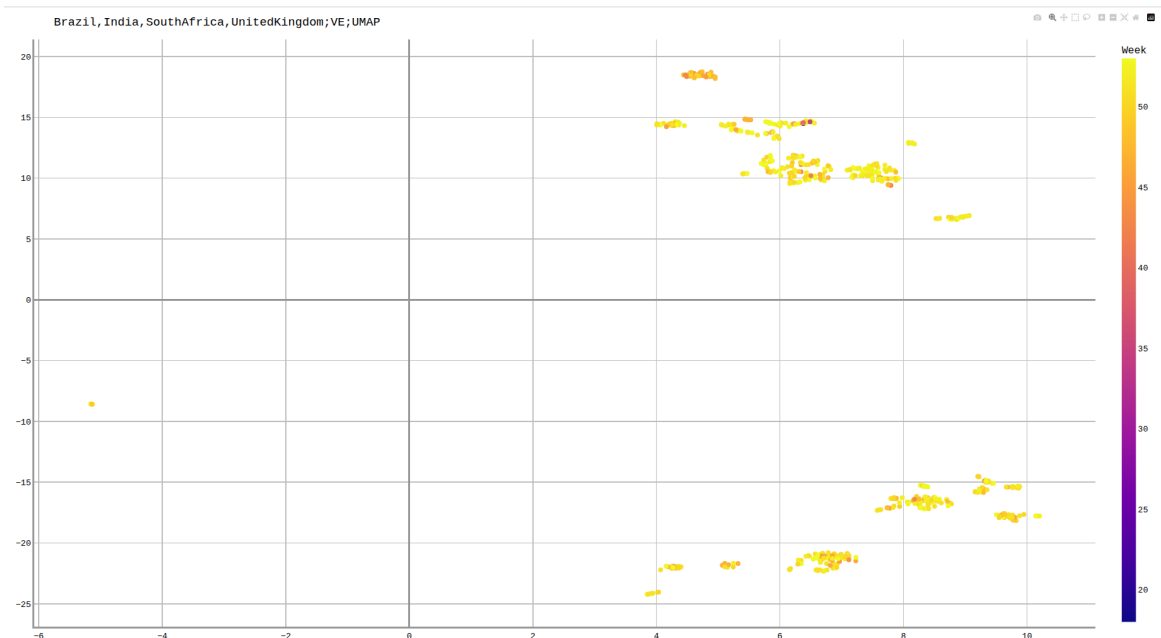

(a)

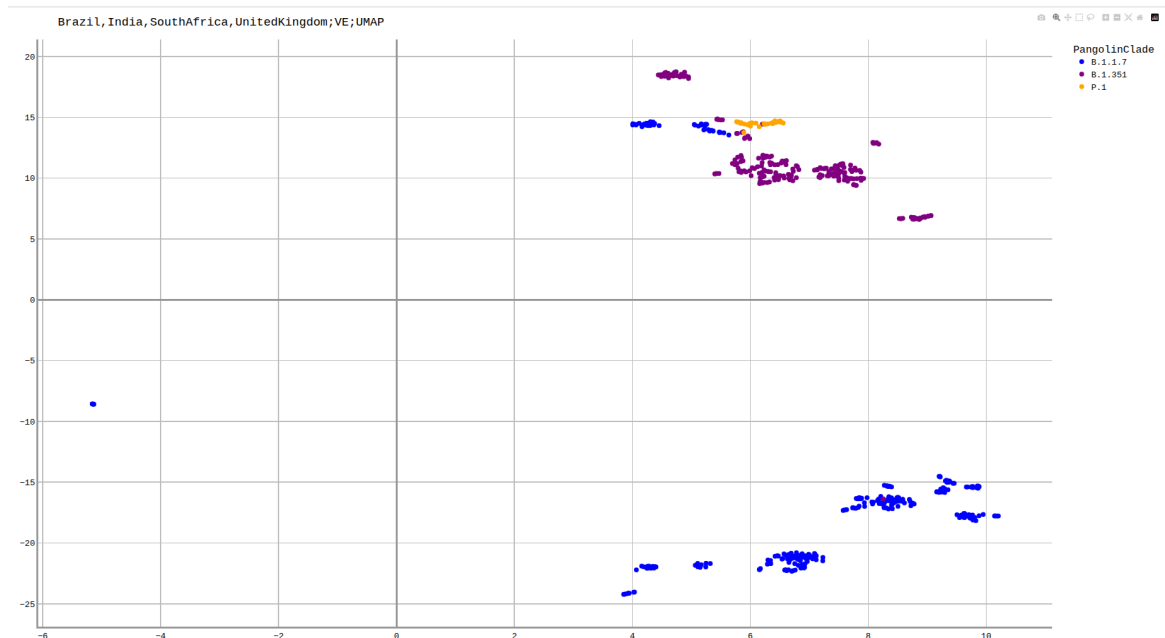

(b)

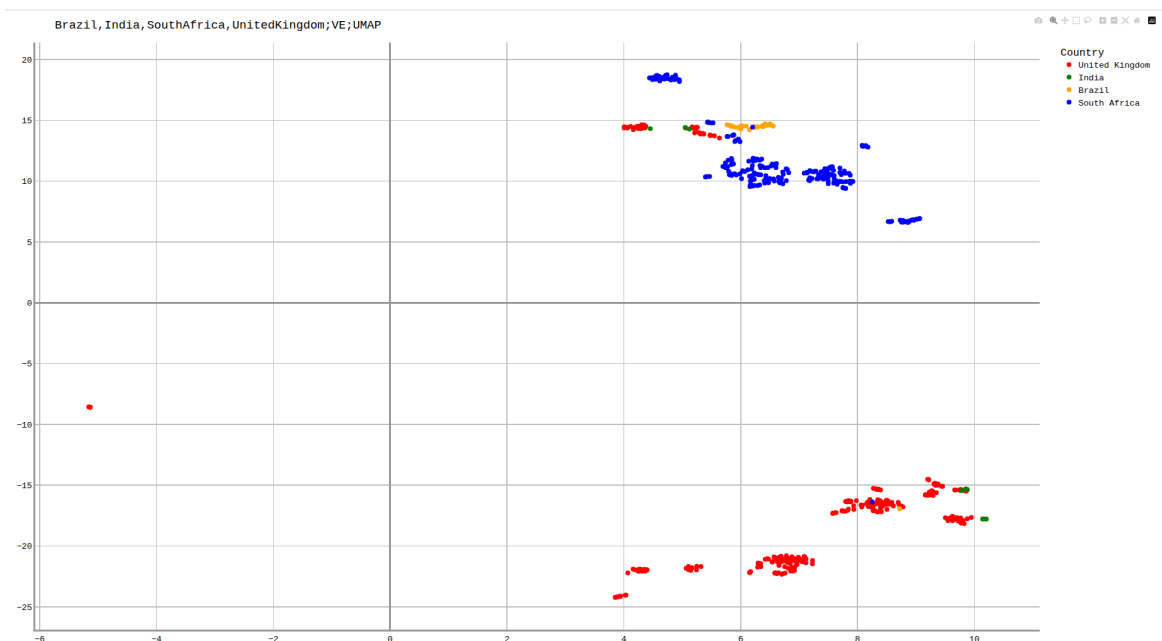

(c)

Figure 1: UMAP visualization of VOC strains from Brazil, India, South Africa and United Kingdom for the year 2020. Color coding is done with respect to (a) Temporal, (b) Pangolin Clade and (c) Geography

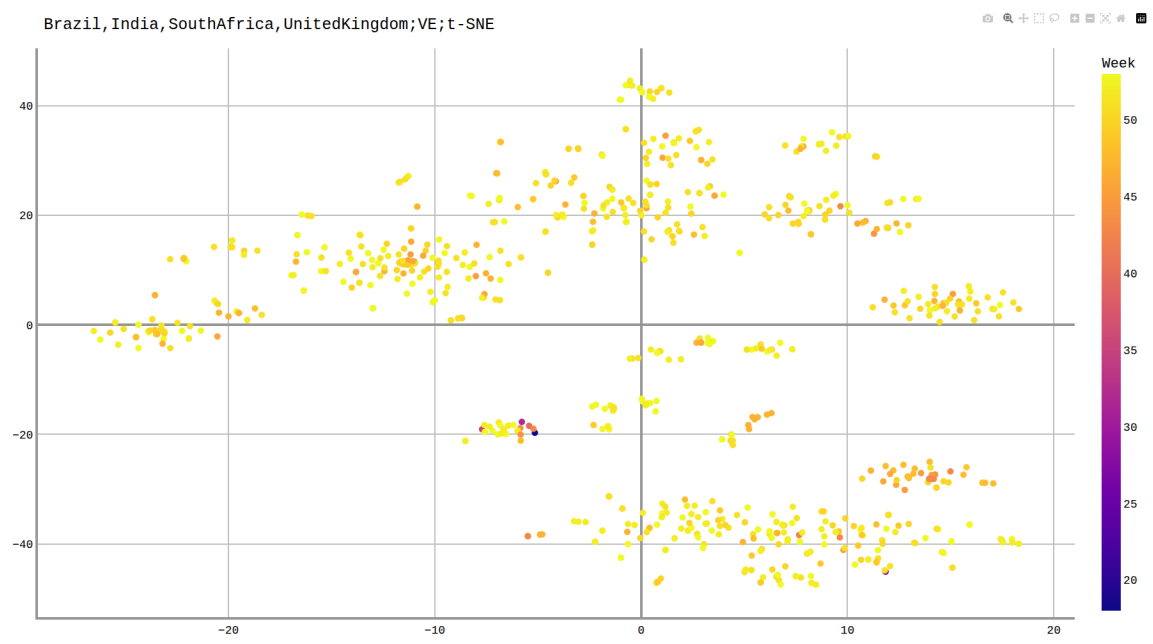

(a)

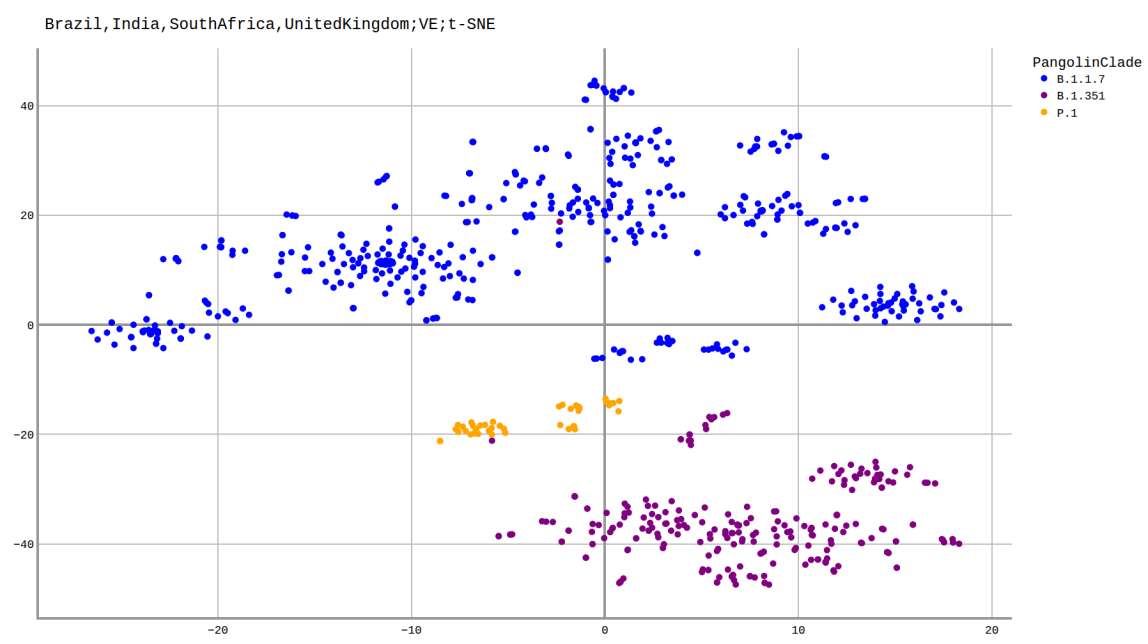

(b)

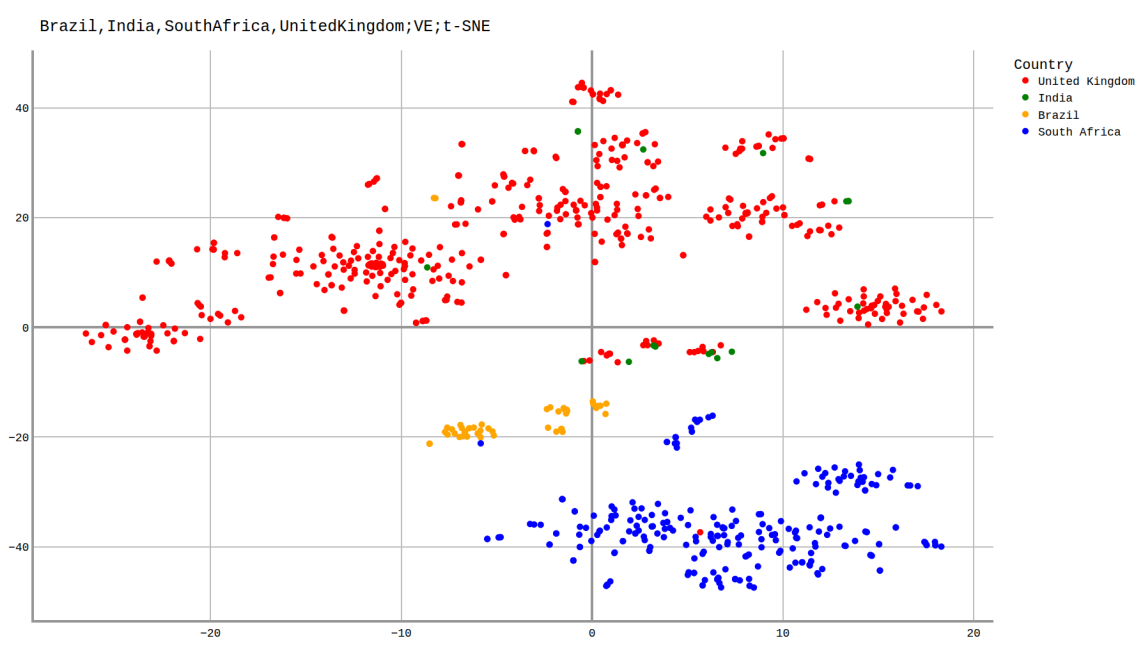

(c)

Figure 2: t-SNE visualization of VOC strains from Brazil, India, South Africa and United Kingdom for the year 2020. Color coding is done with respect to (a) Temporal, (b) Pangolin Clade and (c) Geography

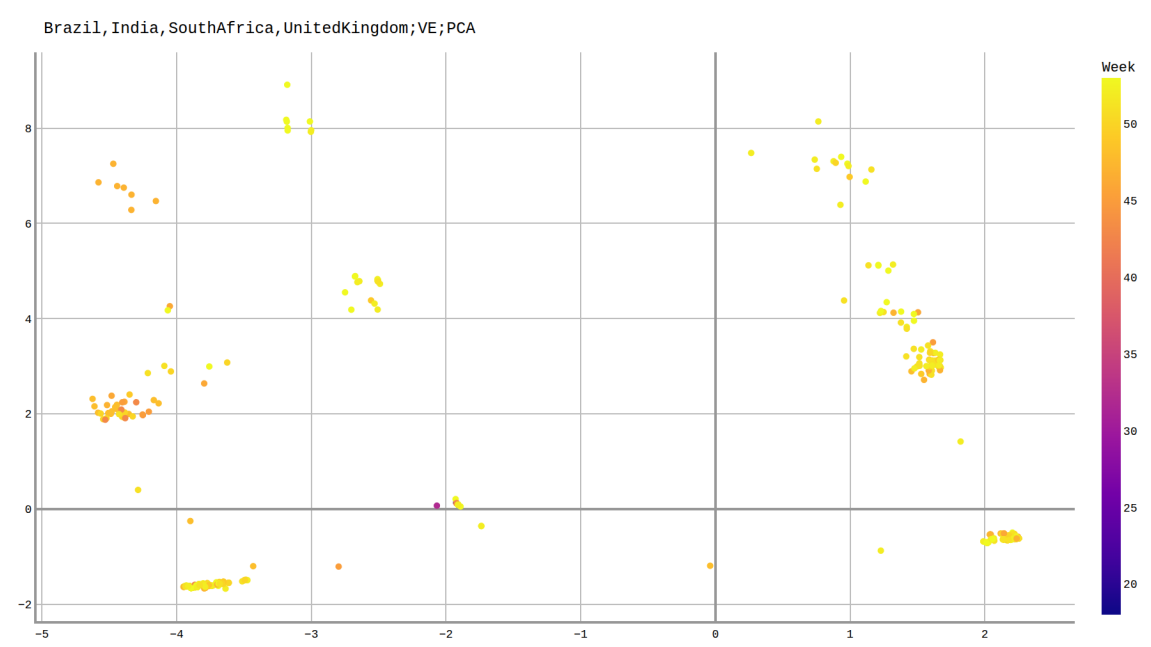

(a)

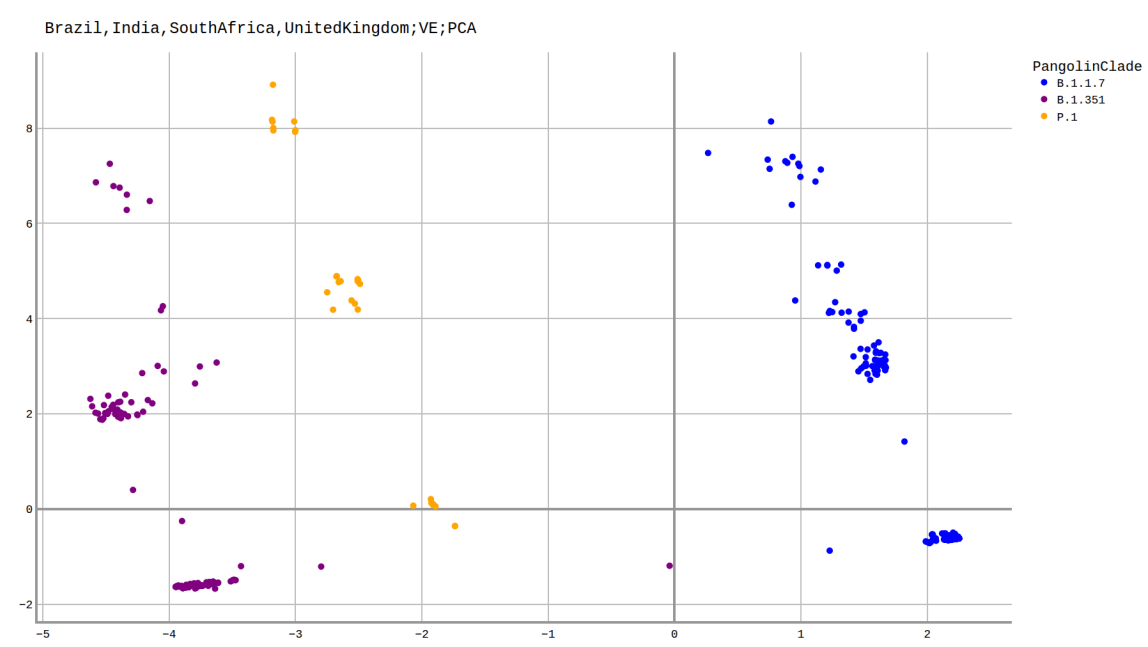

(b)

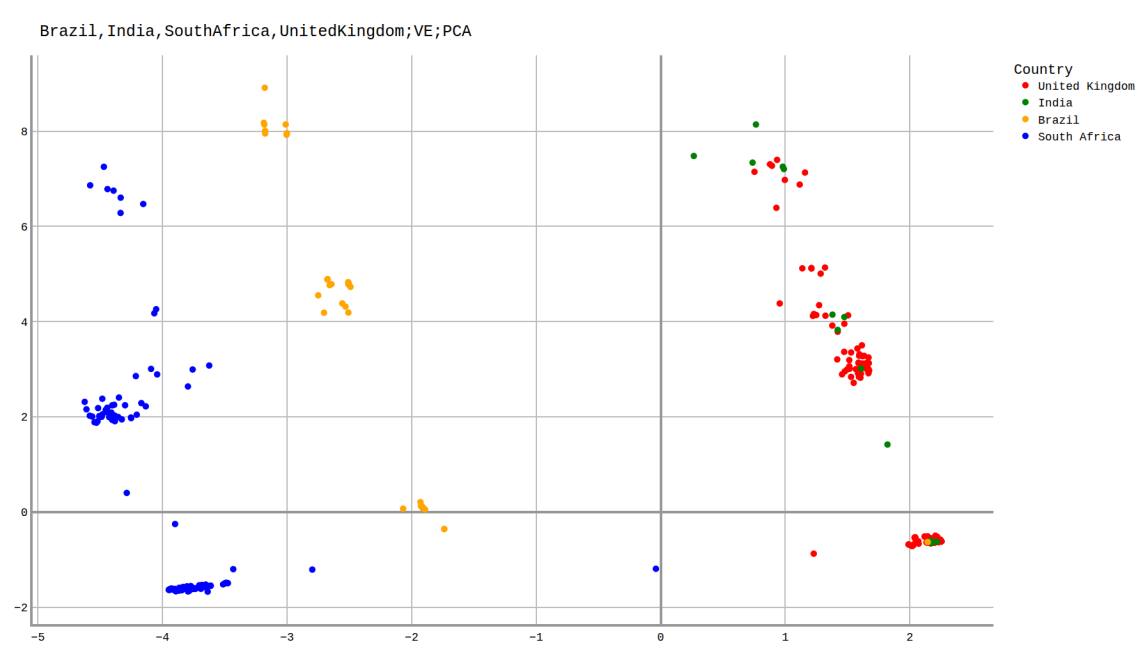

(c)

Figure 3: PCA visualization of VOC strains from Brazil, India, South Africa and United Kingdom for the year 2020. Color coding is done with respect to (a) Temporal, (b) Pangolin Clade and (c) Geography

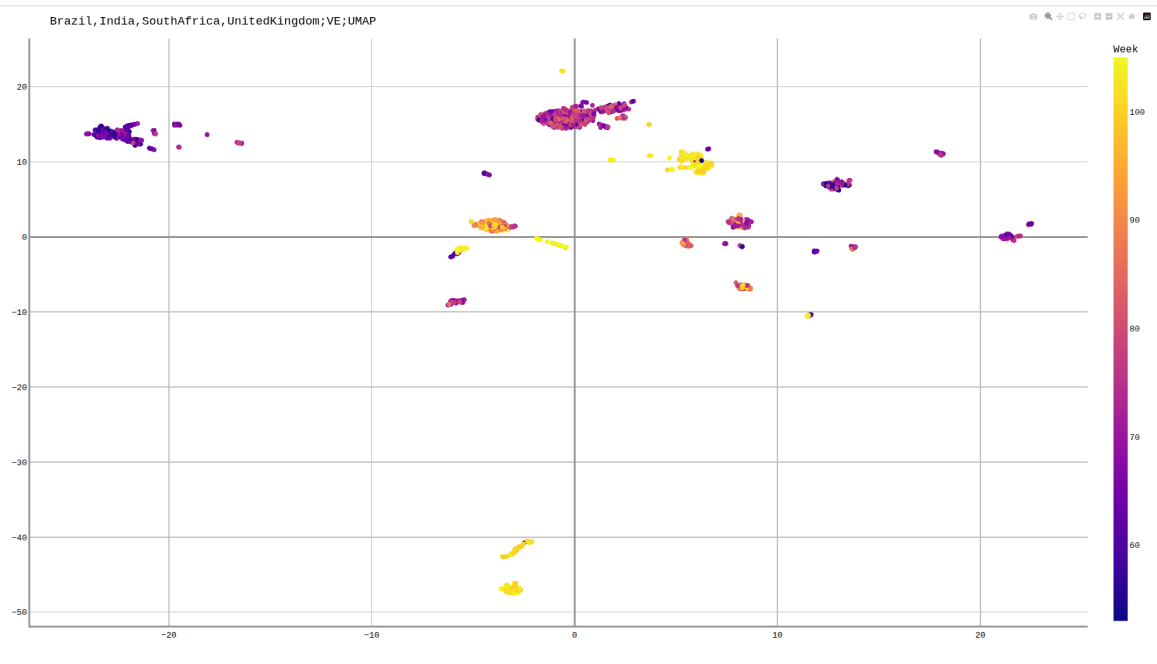

(a)

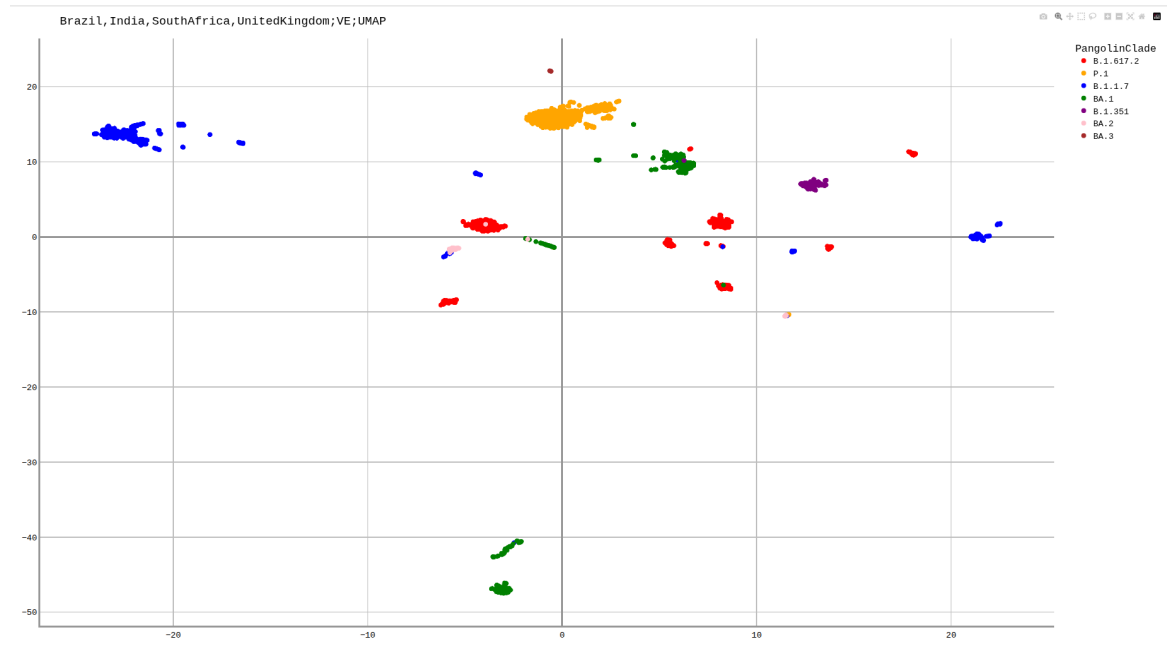

(b)

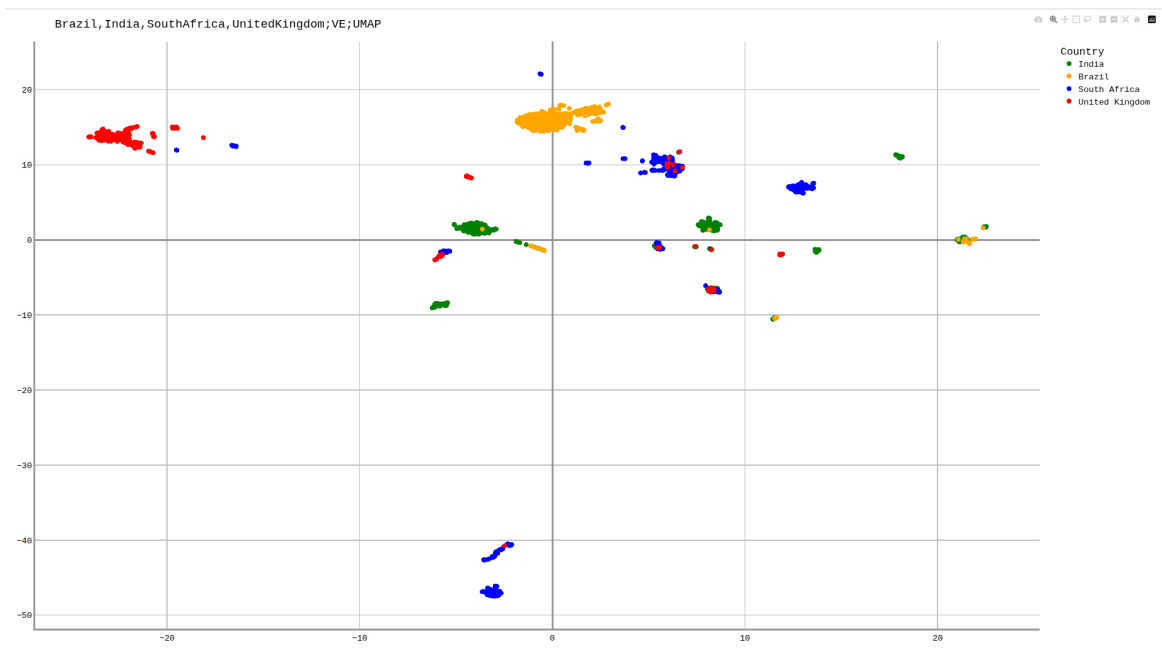

(c)

Figure 4: UMAP visualization of VOC strains from Brazil, India, South Africa and United Kingdom for the year 2021. Color coding is done with respect to (a) Temporal, (b) Pangolin Clade and (c) Geography

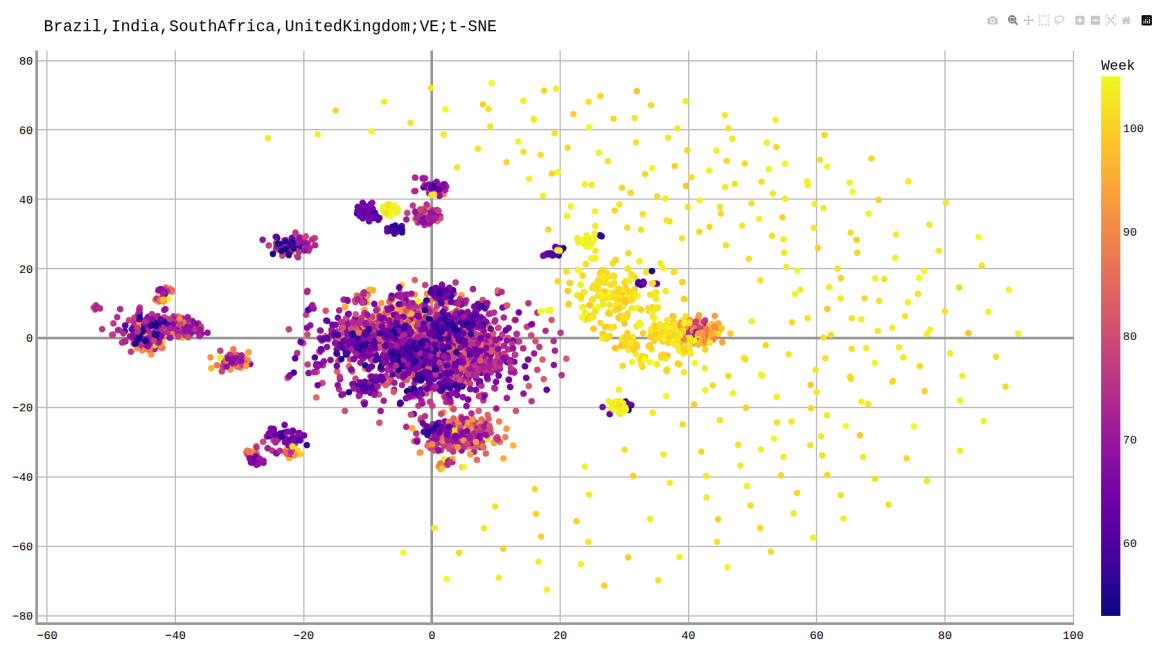

(a)

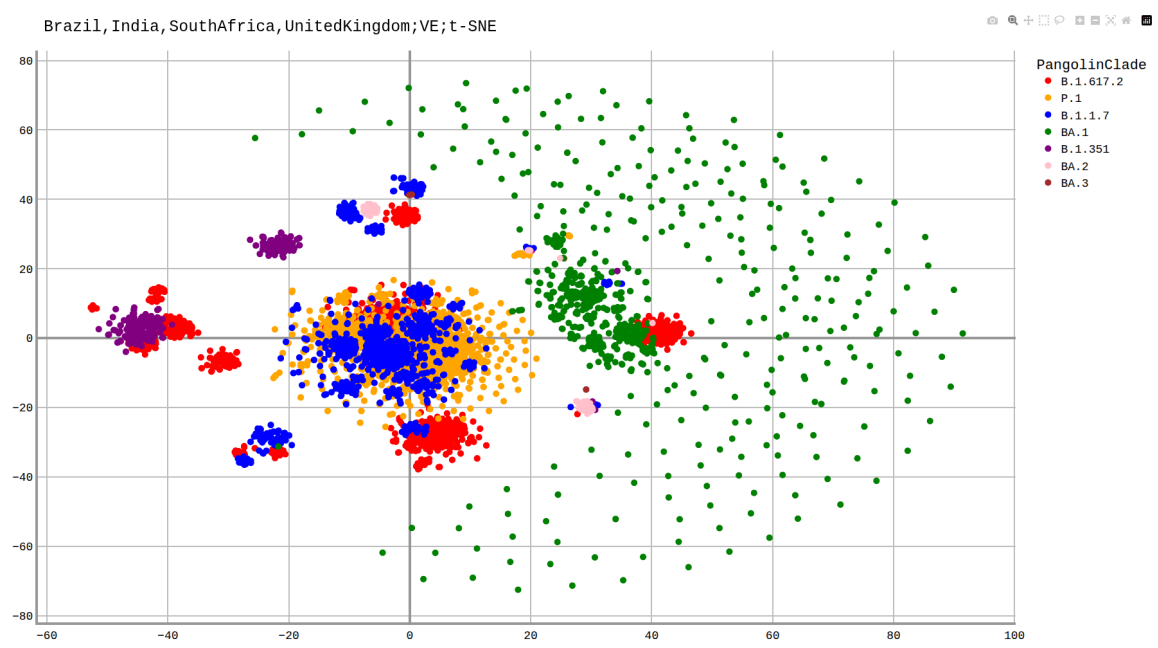

(b)

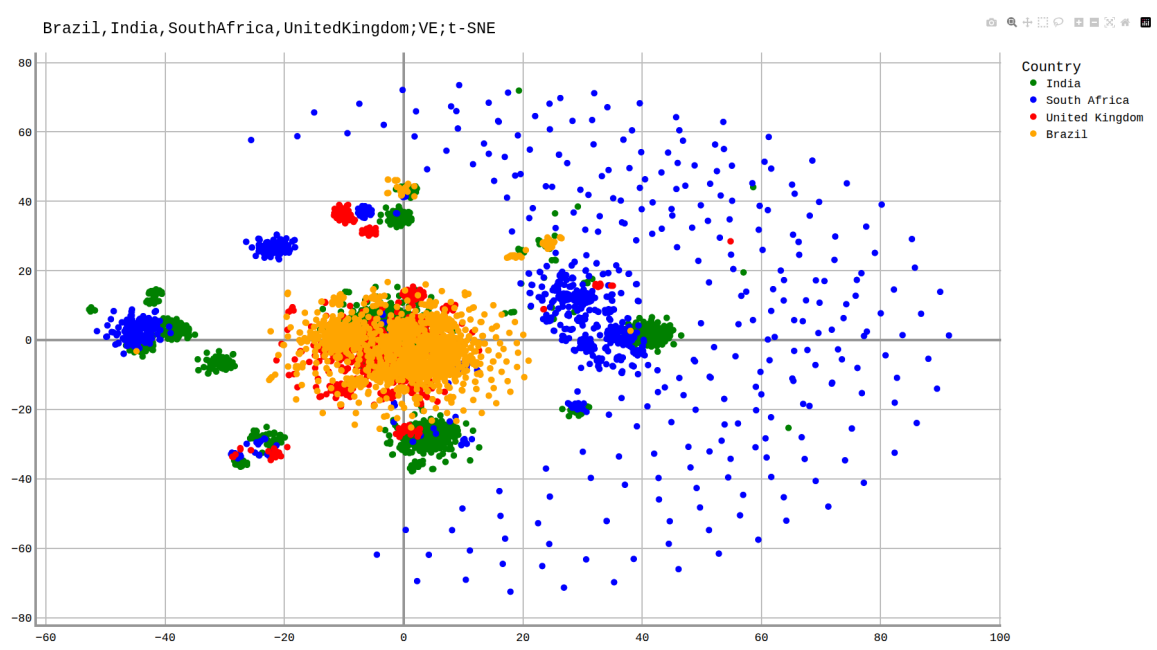

(c)

Figure 5: t-SNE visualization of VOC strains from Brazil, India, South Africa and United Kingdom for the year 2021. Color coding is done with respect to (a) Temporal, (b) Pangolin Clade and (c) Geography

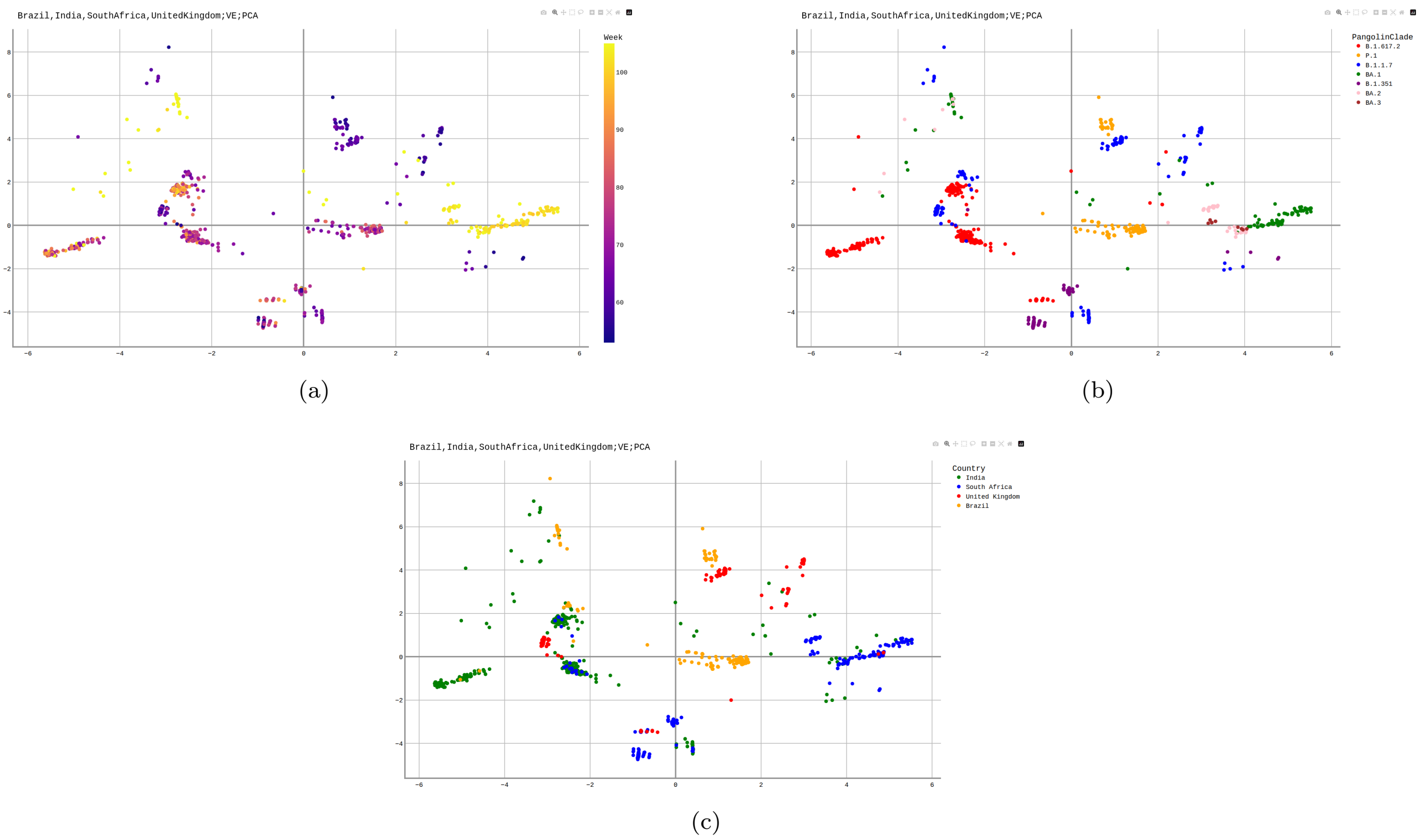

Figure 6: PCA visualization of VOC strains from Brazil, India, South Africa and United Kingdom for the year 2021. Color coding is done with respect to (a) Temporal, (b) Pangolin Clade and (c) Geography

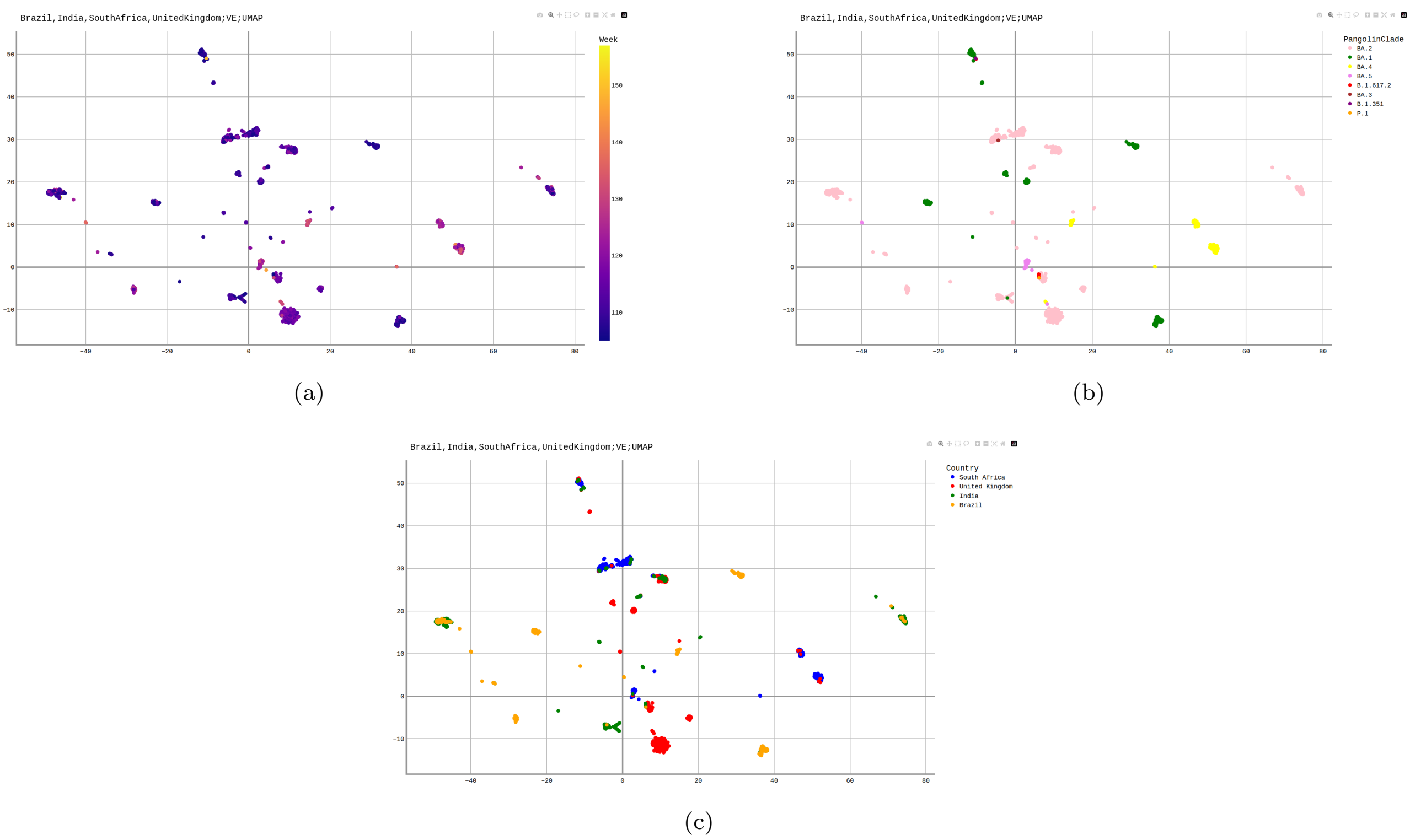

Figure 7: UMAP visualization of VOC strains from Brazil, India, South Africa and United Kingdom for the year 2022. Color coding is done with respect to (a) Temporal, (b) Pangolin Clade and (c) Geography

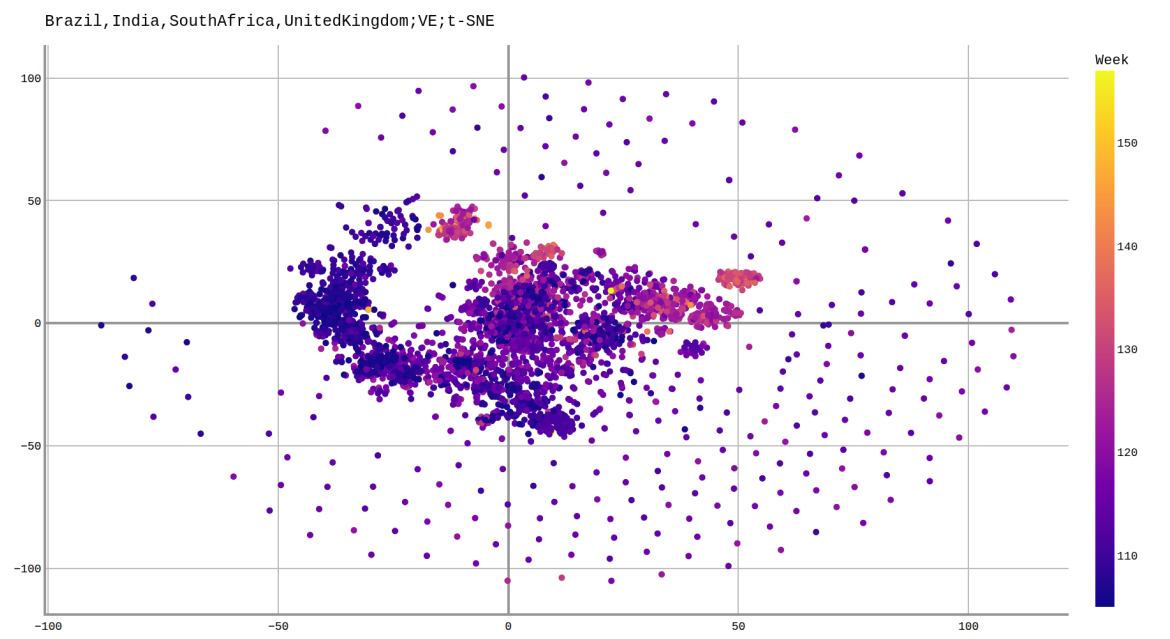

(a)

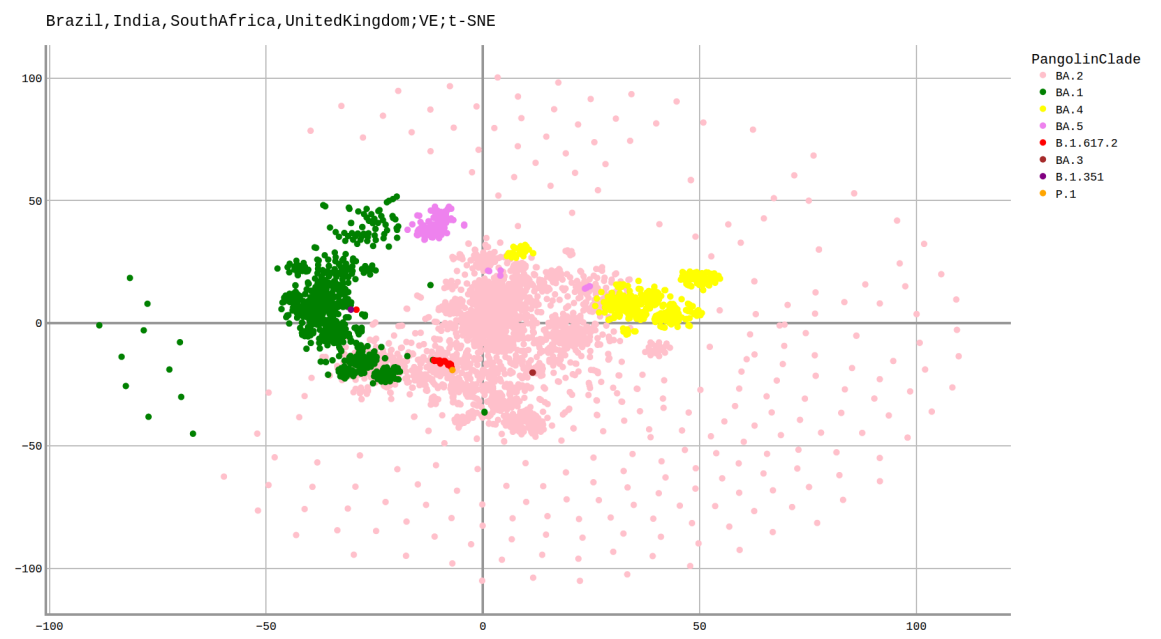

(b)

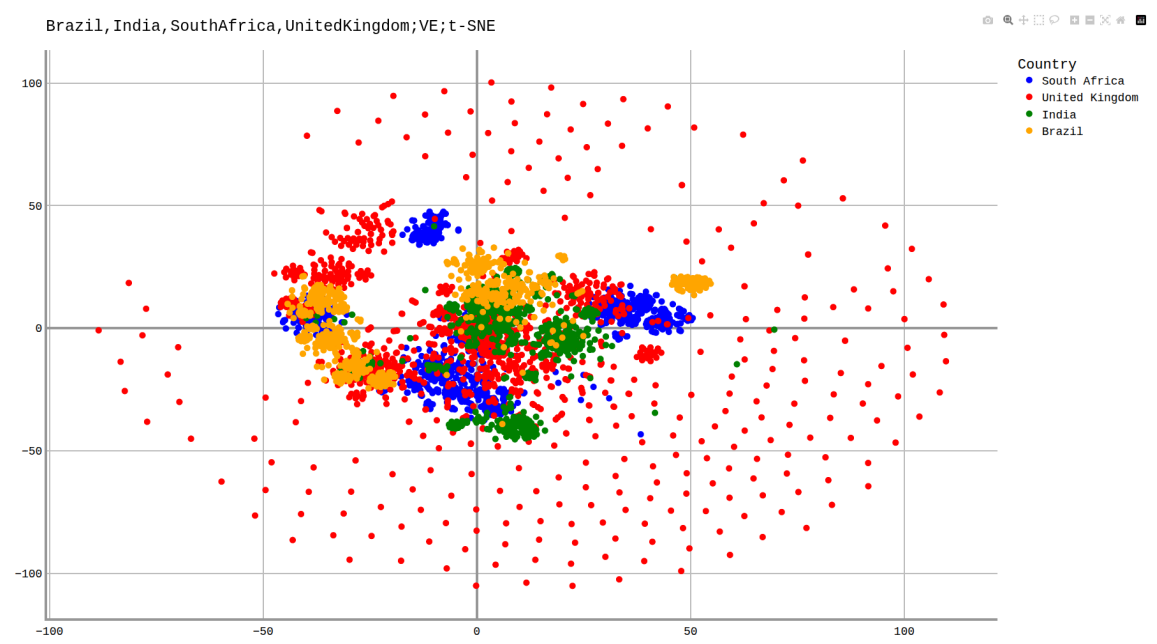

(c)

Figure 8: t-SNE visualization of VOC strains from Brazil, India, South Africa and United Kingdom for the year 2022. Color coding is done with respect to (a) Temporal, (b) Pangolin Clade and (c) Geography

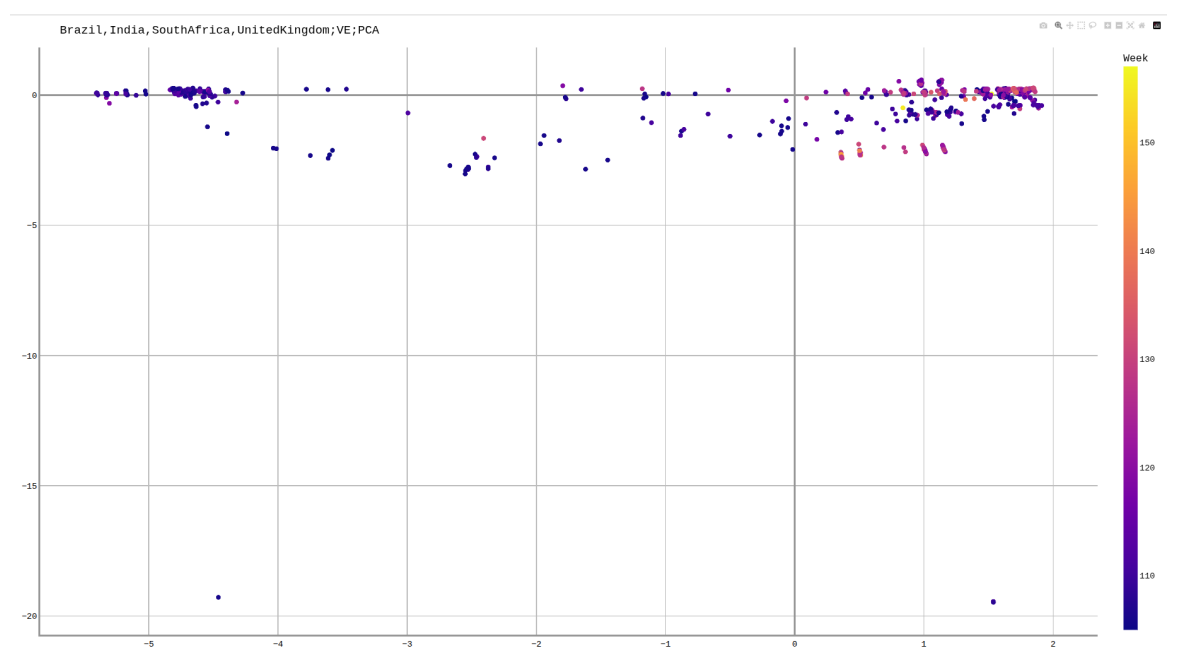

(a)

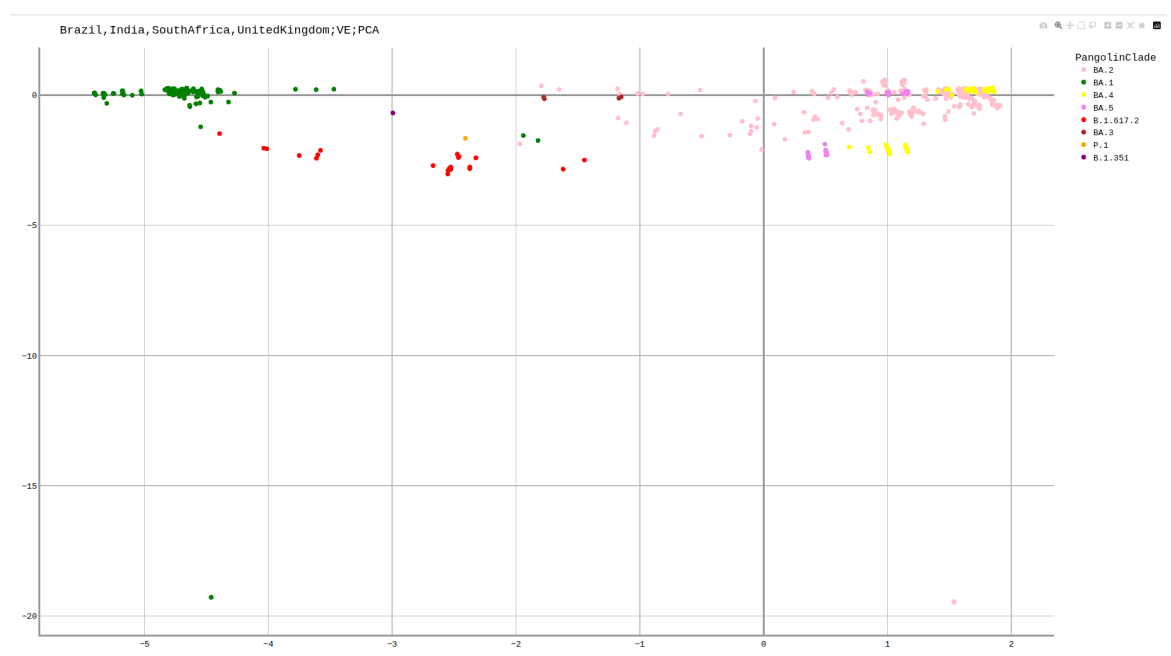

(b)

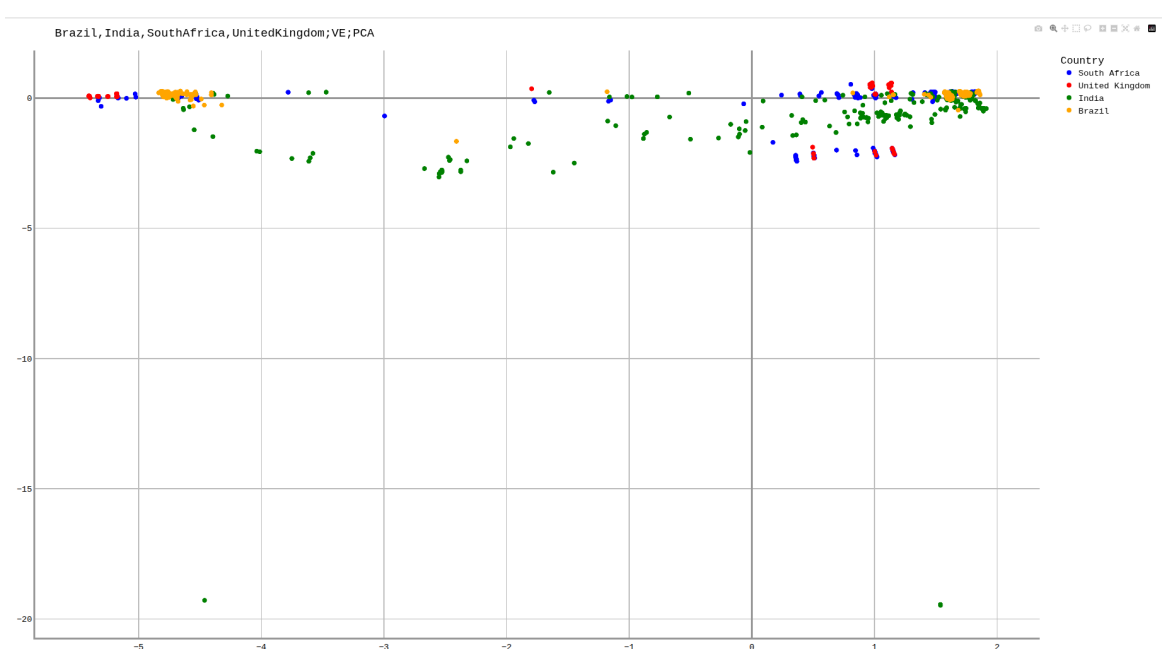

(c)

Figure 9: PCA visualization of VOC strains from Brazil, India, South Africa and United Kingdom for the year 2022. Color coding is done with respect to (a) Temporal, (b) Pangolin Clade and (c) Geography

C Effect of Hyperparameters

Table 1: Hyperparameters and performance across runs for different methods (Saturn scores (mean ± standard deviation) across 10 folds for each method and run)

Run	BOVIZ	PCA_RSP	KPCA	KPCA-RSP	UMAP	T_SNE
**Run-1**	**0.3607**±0.0253	0.1127±0.0295	0.3394±0.0303 Kernel : precomputed (jaccard)	0.1127±0.0295 Kernel : linear	0.2381±0.074 Neigh : 20 min_dis : 0.99 Metric : cosine)	0.2617±0.0451 Perplexity : 20; lr: auto; Metric : jaccard)
**Run-2**	0.0860±0.0192	0.0798±0.008	0.1040±0.00931 Kernel : precomputed (jaccard)	0.0798±0.008 Kernel : linear	**0.1171**±0.0408 Neigh: 100 min_dist : 0.25 metric : cosine	0.0638±0.0229 Perplexity : 20 lr: 200 Metric : cosine
**Run-3**	**0.1582**±0.0159	0.0013±0.0005	0.0469±0.01692 Kernel : linear	0.0350±0.0005 Kernel : precomputed (jaccard)	0.007±0.0079 Neigh : 50 min_dist : 0.25 Metric : jaccard	0.0083±0.0025 Perplexity : 20 lr: 500 Metric : jaccard
**Run-4**	**0.1663**±0.0285	0.0576±0.0	0.0527±0.0 Kernel : linear	0.0589±0.0 Kernel : precomputed (jaccard)	0.0578±0.00572 Neigh : 100 min_dist : 0.5 metric : jaccard	0.0854±0.0039 Perplexity : 20 lr: 300 Metric : euclidean
**Run-5**	**0.0440**±0.0186	0.0206±0.00242	0.0364±0.0048 Kernel : precomputed	0.03±0.0031 Kernel : precomputed (jaccard)	0.0378±0.01512 Neigh : 20 min_dist : 0.5 metric : cosine	**0.0447**±0.0112 Perplexity : 30 lr: 500 Metric : jaccard

Table 2: Summary of conserved variant patterns and biological interpretation across representative clusters

**Cluster**	**Key conserved variants**	**Variant pattern**	**Biological interpretation**
Cluster 5	Spike: L452R, F486V, P681H	Highly conserved Spike-driven signature with low external overlap	Consistent with a coherent Omicron-associated adaptive regime; mutations linked to antibody escape and spike processing
Cluster 7	Spike: G339D, N501Y Non-Spike: ORF1ab:P4715L, N:R203K/G204R	Stable combination of Spike and non-Spike variants	Suggests conserved genomic backbones supporting lineage persistence beyond Spike-driven immune escape
Cluster 0	Background: S:D614G, ORF1ab:P4715L, N:R203K/G204R Spike: A67V, T95I (variable)	Heterogeneous and inconsistently conserved Spike variants	Reflects a more mixed or transitional mutational pattern rather than a single dominant adaptive regime
